# Facet‐Dependent Activity of Fe_3_S_4_ Crystal in Peroxydisulfate Activation for Organic Pollutants Degradation

**DOI:** 10.1002/advs.76644

**Published:** 2026-07-17

**Authors:** Jiaqu Tan, Min Yu, Jinfeng Wang, Dongya Li, Fan Yang, Yulong Zhang, Xueming Lin

**Affiliations:** ^1^ College of Natural Resources and Environment South China Agricultural University Guangzhou People's Republic of China; ^2^ Guangdong Research Center For Agricultural Soil Pollution Prevention and Control Engineering Technology College of Natural Resources and Environment South China Agricultural University Guangzhou People's Republic of China; ^3^ Guangdong Provincial Key Laboratory of Agricultural & Rural Pollution Abatement and Environmental Safety Guangzhou People's Republic of China; ^4^ Key Laboratory of Arable Land Conservation (South China) MOA College of Natural Resources and Environment South China Agricultural University Guangzhou Guangdong People's Republic of China; ^5^ State Key Laboratory of Pollution Control and Resource Reuse School of the Environment Nanjing University Nanjing People's Republic of China; ^6^ School of Environmental Engineering Wuhan Textile University Wuhan People's Republic of China; ^7^ School of Electronic and Electrical Engineering Wuhan Textile University Wuhan People's Republic of China

**Keywords:** facet engineering, Fe_3_S_4_ crystal, Fe−S length, iron cycling, peroxydisulfate

## Abstract

Iron sulfides (Fe_x_S_y_) possess excellent capacities for activating peroxydisulfate (PDS) to degrade organic pollutants from wastewater owing to their highly efficient circulation of Fe(III)/Fe(II), but the intrinsic facet‐activity relations are still unclear to date. Herein, we employed the FLO‐Fe_3_S_4_ with a flower‐like structure and exposed [001] facet, and OCT‐Fe_3_S_4_ with octahedral morphology and exposed [1‐21] facet as catalysts for activating PDS to degrade clothianidin (CLO). Results show that FLO‐Fe_3_S_4_ exhibits significantly enhanced catalytic activity, with a CLO degradation rate of 0.4270 min^−1^, which is 4.4 times higher than that of OCT‐Fe_3_S_4_. The superior reactivity of FLO‐Fe_3_S_4_ can be attributed to its longer Fe−S bonds, making them more prone to breaking and releasing more Fe ions for boosting homogeneous PDS activation. Moreover, the shorter Fe−S bond of OCT‐Fe_3_S_4_ alleviates Fe dissolution, thereby enhancing its catalytic stability and heterogeneous catalytic reaction. Theoretical simulations reveal that the [001] facet of FLO‐Fe_3_S_4_ favors the adsorption of PDS and provides more electrons to decompose PDS compared with the [1‐21] facet of OCT‐Fe_3_S_4_. Overall, this work delves into the intrinsic facet‐activity relations for Fe_3_S_4_ crystals on PDS activation and further unravels the overlooked role of crystal Fe−S length on catalytic reaction.

## Graphical abstract

Fe_3_S_4_ crystal with the exposed [001] facet exhibits higher catalytic activity for activating peroxydisulfate (PDS) to degrade clothianidin (CLO), which can be attributed to the dissolved Fe‐induced homogeneous catalytic process; while Fe_3_S_4_ crystal with the exposed [1‐21] facet exhibits excellent catalytic stability and durability, thereby boosting the heterogeneous PDS activation.

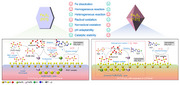

## 1 Introduction

Advanced oxidation processes utilizing peroxydisulfate (PDS‐AOPs) have gained recognition as promising strategies for degrading recalcitrant organic pollutants in wastewater by generating multiple reactive oxygen species (ROS) with high redox potential, such as sulfate radicals (SO_4_^•−^), hydroxyl radicals (•OH), and singlet oxygen (^1^O_2_) [1, 2, 3, 4]. It is known that heterogeneous metal catalysts stand out over homogeneous catalysts for engineering applications due to their superior catalytic capacities, less sludge production, and broad pH adaptabilities; and thus, the development of highly efficient heterogeneous catalysts has emerged as a pivotal research direction in the field of AOPs [5, 6, 7, 8]. Recently, heterogeneous greigite (Fe_3_S_4_) has received extensive attention in AOPs due to its ample redox sites, sustaining the powerful cycling of Fe(III)/Fe(II) by the reductive S species on the catalyst surface [9, 10]. It should be mentioned that Fe_3_S_4_ crystals exhibit distinct morphologies (such as flower‐like and octahedral structures) and exposed facets through different synthesis methods and ingredients, but both of them can efficiently activate PDS or peroxymonosulfate (PMS) to degrade organic pollutants, mainly owing to their significantly promoted circulation of Fe(III)/Fe(II) and generation of abundant ROS [11, 12]. Compared with other Fe‐based catalysts, like Fe^0^ [13], and FeOOH [14], the existence of abundant reductive S sites on Fe_3_S_4_ crystal enables efficient iron cycling and maintains stable ROS generation, showing great potential for wastewater treatment.

At present, numerous reports have indicated that the activity of a metal catalyst is closely related to its structure and exposed facet [15, 16, 17, 18, 19]. For example, Wang et al. discovered that the Co_3_O_4_ crystal with exposed [001] facet exhibits the highest activity on activating PMS compared to the [111] and [112] facets, mainly because the [001] facet can lower the energy barrier of the key step during ^1^O_2_ production process [20]. Zheng et al. also found that the facet engineering of BiVO_4_ can significantly enhance PDS activation for contaminant degradation [21]. In addition to the exposed facet, it is reported that the shape of the catalyst can greatly affect its activity, e.g., octahedral Mn_3_O_4_ exhibits the highest reactivity for PMS activation compared with the plate and cube shapes [22]. Such findings strongly confirm that the exposed facet is of vital importance for the catalytic activity of a catalyst. In addition, metal catalysts with distinct exposed facets may leach different concentrations of metal ions during reaction, which likely affect the catalytic pathways and mechanisms, but few researchers pay attention to exploring that. To date, the detailed mechanisms on how the morphology and exposed facet of Fe_3_S_4_ crystal, the length of Fe─S bond, and the amount of Fe dissolution from Fe_3_S_4_ crystal affect the PDS activation performance have not been well elucidated.

Hence, the overall goal of this work is to identify the PDS activation mechanisms on Fe_3_S_4_ crystals with specific exposed facets and provide deep insights on facet‐dependent ROS generation behaviors. Based on the catalytic experiments, we discovered that the distinct PDS activation mechanisms by FLO‐Fe_3_S_4_ with a flower‐like structure and exposed [001] facet, and OCT‐Fe_3_S_4_ with octahedral morphology and exposed [1‐21] facet were mainly attributed to the length of Fe−S and the amount of Fe dissolution. In detail, FLO‐Fe_3_S_4_ demonstrates higher activity and outstanding pH adaptability for PDS activation to degrade clothianidin (CLO), which can be attributed to its longer Fe−S bonds, making it more prone to breaking and releasing massive Fe ions (∼10 mg/L) for boosting homogeneous PDS activation. While OCT‐Fe_3_S_4_ exhibits shorter Fe−S bonds, which can alleviate the Fe dissolution (∼0.7 mg/L), thereby enhancing its catalytic stability and heterogeneous catalytic reaction. Further theoretical calculations suggest that the [001] facet of FLO‐Fe_3_S_4_ favors the adsorption of PDS and provides more electrons to decompose PDS compared with the [1‐21] facet of OCT‐Fe_3_S_4_. Overall, this work not only provides novel insights into the facet‐dependent mechanisms of PDS activation over Fe_3_S_4_ crystals but also unravels the overlooked role of crystal Fe−S length on catalytic reaction.

## 2 Results and Discussion

### 2.1 Structural Characterization

**FIGURE 1:**
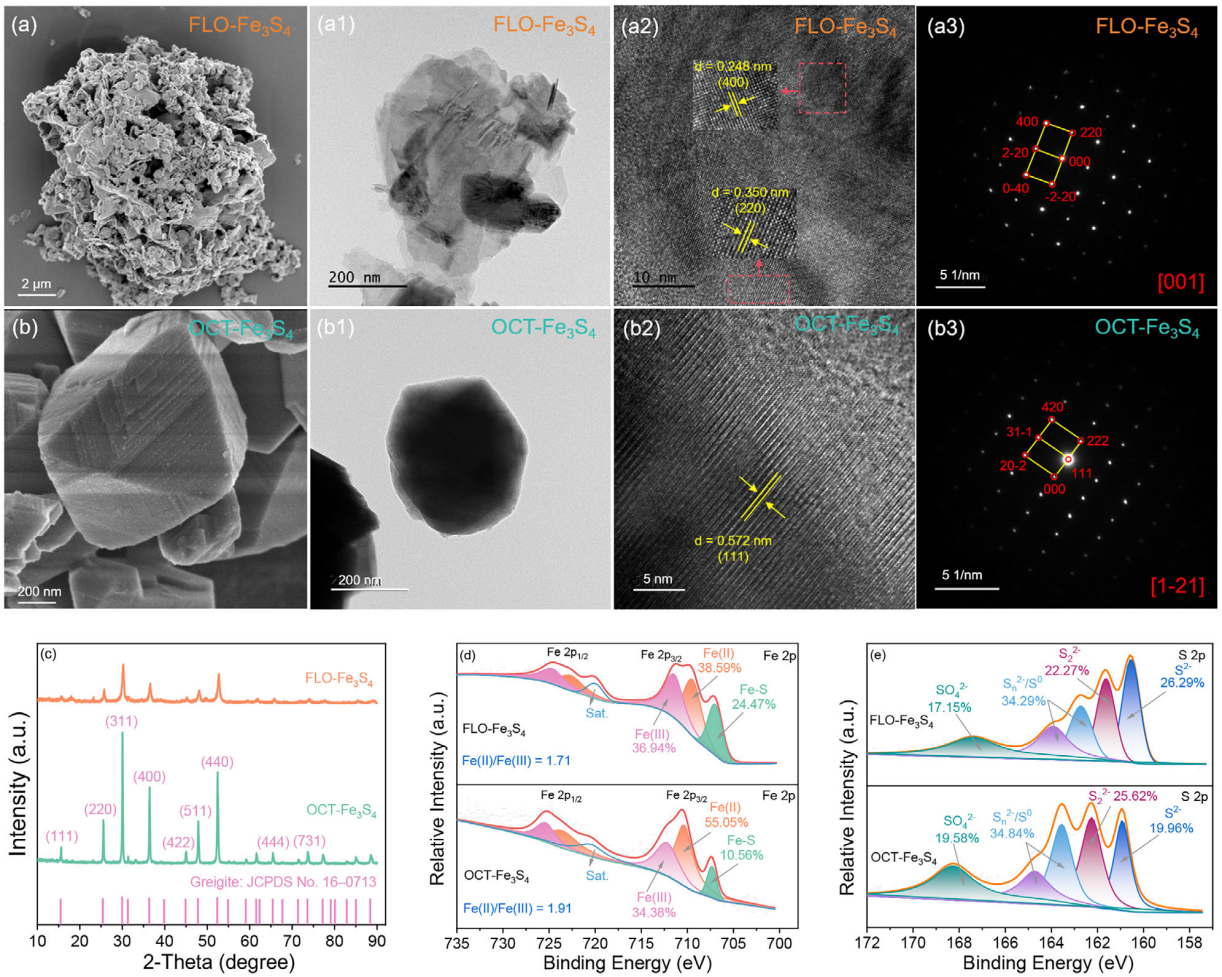
(a, b) SEM images, (a1, b1) TEM images, (a2, b2) HRTEM images, (a3, b3) SAED patterns, and (c) XRD patterns of FLO‐Fe_3_S_4_ and OCT‐Fe_3_S_4_. High‐resolution XPS spectra of (d) Fe 2p and (e) S 2p for FLO‐Fe_3_S_4_ and OCT‐Fe_3_S_4_.

The structures of resulting Fe_3_S_4_ crystals were first characterized by scanning electron microscope (SEM). As depicted in Figure 1a,b, FLO‐Fe_3_S_4_ is assembled by numerous nanosheets and exhibits a well‐defined flower‐like structure with an average length of ∼15 µm, while OCT‐Fe_3_S_4_ manifests a specific octahedral structure with sizes of ∼500 nm. High‐resolution transmission electron microscope (HRTEM) images and selected‐area electron diffraction (SAED) patterns in Figure 1a1‐a3 show that FLO‐Fe_3_S_4_ exhibits distinct (200) and (400) crystal planes with lattice spacings of 0.350 and 0.248 nm, respectively, demonstrating that FLO‐Fe_3_S_4_ is grown with the [001] orientation and the preferentially exposed facet is the [001] facet. For OCT‐Fe_3_S_4_ crystal (Figure 1b1‐b3), a (111) plane with a lattice spacing of 0.572 nm is observed along the [1‐21] zone axis, indicative of its predominant exposure of [1‐21] facets.

X‐ray diffraction (XRD) patterns in Figure 1c show that all samples exhibit virtually identical diffraction profiles, with characteristic peaks at 25.4°, 29.9°, 36.3°, 47.8°, and 52.3° corresponding to the (220), (311), (400), (511), (222), (400) and (440) crystallographic planes of cubic Fe_3_S_4_ (JCPDS No. 16–00713), respectively [23]. It is observed that all the diffraction peak intensities of OCT‐Fe_3_S_4_ are much stronger than those of FLO‐Fe_3_S_4_, indicating its purer crystalline phase and higher crystallinity [20]. As revealed in electron paramagnetic resonance (EPR) spectra (Figure S1), both FLO‐Fe_3_S_4_ and OCT‐Fe_3_S_4_ show characteristic signals for sulfur vacancies (SVs) at the g value of 2.003, wherein FLO‐Fe_3_S_4_ exhibits a more intense signal, suggesting its higher density of SVs [24, 25, 26, 27]. X‐ray photoelectron spectroscopy (XPS) analysis is shown in Figure S2, from the Fe 2p spectra (Figure 1d), the splitting peaks at 707.3, 709.5, and 711.5 eV can be ascribed to the Fe(II)‐S, Fe(II), and Fe(III) species, respectively [23], and the ratio of Fe(II)/Fe(III) follows the order of OCT‐Fe_3_S_4_ (1.91) > FLO‐Fe_3_S_4_ (1.71). The inductively coupled plasma optical emission spectroscopy (ICP‐OES) analysis indicates that OCT‐Fe_3_S_4_ exhibits a higher weight percentage of Fe compared with FLO‐Fe_3_S_4_ (50% vs. 40%) (Figure S3). As for the S 2p spectra (Figure 1e), the peaks at 161.5, 162.6, and 168.2 eV can be assigned to the apical sulfide ligands (S^2−^), bridging disulfides (S_2_^2−^), and sulfates (SO_4_^2−^), respectively [8, 28]. While the peaks at 163.4 and 164.8 eV belong to polysulfides (S_n_^2−^) or sulfur (S^0^), since their peaks usually occur together and overlap, making differentiation difficult. It is worth noting that FLO‐Fe_3_S_4_ has a relatively higher low‐valent sulfur content (S^2−^ and S_2_^2−^) compared with OCT‐Fe_3_S_4_ (Figure S4), which may be more conducive to the regeneration of Fe(II), thereby promoting the catalytic process. Figure S5 illustrates that the O 1s spectra can be deconvolved into lattice oxygen (Fe−O, 530.2 eV), lattice hydroxyl (Fe−OH, 531.8 eV), and adsorbed H_2_O (533.3 eV), wherein the existence of lattice oxygen can facilitate PDS activation and ROS generation [28, 29]. Additionally, FLO‐Fe_3_S_4_ demonstrates a higher BET surface area of 6.98 m^2^/g compared to the OCT‐Fe_3_S_4_ (3.08 m^2^/g) (Figure S6), which is possibly attributed to the flake structure of FLO‐Fe_3_S_4_ preventing crystal restacking and optimizing pore distribution. A larger surface area may improve porosity, enhance active site exposure and mass transport, thereby increasing the catalytic activity [30]. From vibrating sample magnetometer (VSM) analysis (Figure S7), both of FLO‐Fe_3_S_4_ and OCT‐Fe_3_S_4_ retain high saturation magnetization (53.2 emg/g vs. 34.3 emg/g), indicating that these Fe_3_S_4_ catalysts can be easy to separate and recover from wastewater, benefiting the practical application.

Conclusively, the above results demonstrate the successful synthesis of Fe_3_S_4_ catalysts with distinct shapes and exposed facets, which can serve as a model platform to elucidate the catalyst facet‐dependent PDS activation mechanisms.

### 2.2 Catalytic Performance of the Prepared Fe_3_S_4_ Crystals

**FIGURE 2:**
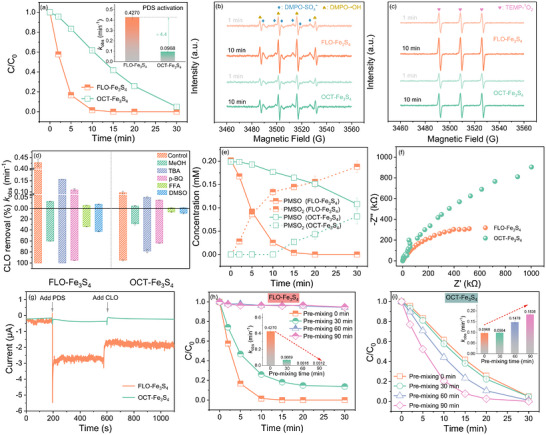
(a) CLO degradation by PDS activation on FLO‐Fe_3_S_4_ and OCT‐Fe_3_S_4_. Spin‐trapping EPR signals for (b) •OH/SO_4_^•−^ and (c) ^1^O_2_ in different Fe_3_S_4_/PDS systems. (d) Scavenging experiments ([MeOH] = [TBA] = 100 mM, [p‐BQ] = [FFA] = 1 mM, [DMSO] = 2 mM) and (e) PMSO degradation and corresponding PMSO_2_ production in the FLO‐Fe_3_S_4_/PDS and OCT‐Fe_3_S_4_/PDS systems. (f) EIS Nyquist plots and (g) chronoamperometric i‐t curves of different systems. (h‐i) Effects of pre‐mixing experiment on the CLO degradation in the FLO‐Fe_3_S_4_/PDS and OCT‐Fe_3_S_4_/PDS systems. Reaction conditions: [Catalysts] = 0.1 g/L, [PDS] = 1.0 mM, initial pH 3.40, [CLO] = 5 mg/L, and T = 25°C.

To systematically evaluate the effects of different exposed facets on catalytic activities, PDS activation over FLO‐Fe_3_S_4_ and OCT‐Fe_3_S_4_ was investigated using the degradation of CLO as a model reaction. Results show that direct catalyst adsorption or PDS oxidation only accounted for ∼7% of CLO removal (Figure S8), but complete CLO degradation was achieved within 30 min with the introduction of FLO‐Fe_3_S_4_ into PDS/CLO mixtures, underscoring the pivotal role of the catalytic oxidation process (Figure 2a). Kinetic analysis shows that FLO‐Fe_3_S_4_ exhibited superior reactivity on PDS activation, as substantiated by its degradation rate constants (*k*_obs_) of 0.4270 min^−1^, which was approximately 4.4 times higher than that of OCT‐Fe_3_S_4_ (0.0968 min^−1^). Consistently, FLO‐Fe_3_S_4_ decomposed 73% of PDS over a 30‐min catalytic reaction, significantly outperforming the reactivity of OCT‐Fe_3_S_4_ (20%) (Figure S9). The exceptional catalytic performance of FLO‐Fe_3_S_4_ can also be evidenced by comparing with other recently reported transition metal catalysts, as detailed in Table S1 and Figure S10. The effects of catalyst and PDS dosages on CLO degradation in the FLO‐Fe_3_S_4_/PDS system were investigated in Figure S11, and the optimal reaction parameters were determined to be 0.1 g/L catalyst and 1.0 mM PDS. The effect of temperature was shown in Figure S12, according to the Arrhenius equation based on first‐order kinetics, the reaction activation energy (Ea) values were determined to be 29.48 and 28.66 kJ/mol in the FLO‐Fe_3_S_4_/PDS and OCT‐Fe_3_S_4_/PDS systems, respectively, indicating that the PDS activation over two Fe_3_S_4_ crystals was dominated by intrinsic chemical reactions rather than mass transfer.

To further assess the environmental applicability of the two constructed Fe_3_S_4_‐based catalytic systems, several inorganic anions such as Cl^–^ and NO_3_^–^, and natural organic matter (NOM) were selected to investigate their influences on CLO degradation since they were pervasive in the natural water environment. It can be clearly seen from Figure S13 that the FLO‐Fe_3_S_4_/PDS system remained superior in tolerance under the coexistence of Cl^–^, NO_3_^–^ and NOM, achieving 100% of CLO degradation within 30 min. In comparison, the presence of Cl^–^ and NOM significantly inhibited CLO degradation in the OCT‐Fe_3_S_4_/PDS system. In addition, other pollutants, such as thiamethoxam (THM), imidacloprid (IMI), bisphenol A (BPA), bisphenol S (BPS) and hexafluorobisphenol A (BPAF), the FLO‐Fe_3_S_4_/PDS system exerted excellent elimination performance (Figure S14), but there were significant differences in removing disparate pollutants in the OCT‐Fe_3_S_4_/PDS system, demonstrating that the ROS generated in the FLO‐Fe_3_S_4_/PDS system were more powerful and thus the enhancement of FLO‐Fe_3_S_4_ in pollutant elimination was universal [31]. Therefore, it is concluded that FLO‐Fe_3_S_4_ exhibits superior anti‐interference performance on PDS activation for degrading CLO compared to OCT‐Fe_3_S_4_.

### 2.3 Exploration of the Reaction Mechanisms in Two Fe_3_S_4_/PDS Systems

#### 2.3.1 Determination of Reactive Species

In Figure 2b,c and Figure S15, strong EPR characteristic signals corresponding to DMPO‐SO_4_^•−^, DMPO‐•OH, TEMP‐^1^O_2_, and DMPO‐O_2_^•−^ adducts were detected in two Fe_3_S_4_‐mediated catalytic systems, with their intensities increasing over time, providing compelling evidence that two Fe_3_S_4_ crystals can activate PDS to generate SO_4_^•−^, •OH, O_2_^•−^, and ^1^O_2_. Comparative analysis demonstrated that the intensities of these ROS signals in the FLO‐Fe_3_S_4_/PDS system were much higher at the same reaction time, underscoring its exceptional catalytic activity. Further, the chemical quenching experiments were implemented, in which methanol (MeOH), tert‐butyl alcohol (TBA), p‐benzoquinone (p‐BQ), furfuryl alcohol (FFA) and dimethyl sulfoxide (DMSO) were used to quench SO_4_^•−^ and •OH, •OH, O_2_^•−^, ^1^O_2_ and high‐valent iron‐oxo active species (≡Fe(IV) = O), respectively [32, 33, 34, 35]. For two reaction systems (Figure 2d), addition of FFA resulted in more potent inhibition on CLO degradation, followed by DMSO and MeOH, demonstrating the substantial contribution of ^1^O_2_, ≡Fe(IV) = O and SO_4_^•−^ to CLO degradation. The formation of ≡Fe(IV) = O can be confirmed by the selective sulfoxide‐to‐sulfone oxidation experiment, wherein high methyl phenyl sulfoxide (PMSO)/methyl phenyl sulfone (PMSO_2_) conversion (η, PMSO_2_/PMSO) was obtained in the FLO‐Fe_3_S_4_/PDS and OCT‐Fe_3_S_4_/PDS systems (Figure 2e and Figure S16) [36, 37]. Introduction of TBA and p‐BQ led to a small‐magnitude reduction in CLO degradation rate, indicating the involvement of •OH and O_2_^•−^. Control experiments conducted under a nitrogen atmosphere revealed that the generation of ROS was mainly derived from PDS activation rather than dissolved oxygen, as evidenced by the negligible inhibitory effects on CLO degradation (Figure S17). Subsequently, the relative contributions of aforesaid reactive species to CLO degradation were estimated via the approach provided by Hu et al. [38], as described in Text S6. As a result, the contributions of SO_4_^•−^, •OH, O_2_^•−^, ^1^O_2_ and ≡Fe(IV) = O in the FLO‐Fe_3_S_4_/PDS system were calculated to be 29.38%, 63.17%, 74.41%, 96.75% and 93.13%, respectively (Figure S18). Similar results were observed in the OCT‐Fe_3_S_4_/PDS system, wherein ^1^O_2_ and ≡Fe(IV) = O contributed the most (97.73% and 95.32%). Notably, the proportion sum of each contribution exceeds 100%, possibly due to the interconversion of active species, such O_2_^•−^ and ^1^O_2_. In other word, when O_2_^•−^ was quenched by p‐BQ, the generation of ^1^O_2_ was also inhibited [39]. From these results, the relative significance of these reactive species was found to follow the order ^1^O_2_ ≈ ≡Fe(IV) = O > O_2_^•−^ > •OH > SO_4_^•−^ in the degradation of CLO. However, replacing H_2_O with D_2_O failed to promote CLO degradation in two reaction systems even though the lifetime of ^1^O_2_ in D_2_O (∼68 µs) can be extended by more than 18 times than that in H_2_O (∼3.7 µs), substantiating ^1^O_2_ only acted as an auxiliary role (Figure S17) [40]. It is also reported that ^1^O_2_ scavengers, like FFA, can directly react with the oxidant, potentially leading to misleading conclusions [41].

To delve the role of the electron transfer pathway (ETP) mechanism on CLO degradation, electrochemical analyses were conducted. As displayed in Figure 2f, the electrochemical impedance spectroscopy (EIS) Nyquist plot of FLO‐Fe_3_S_4_ showed a smaller semicircle, signifying its lower electrochemical impedance and superior electron transfer and transmission characteristics than OCT‐Fe_3_S_4_ [42, 43]. Predictably, chronoamperometric measurements demonstrated a sharp increase from −0.4062 to −5.4718 µA in the current of the working electrode for FLO‐Fe_3_S_4_ in response to PDS introduction, which was more pronounced than that observed in OCT‐Fe_3_S_4_ system (Figure 2g). These variations in current reflected the dynamic electron redistribution occurring between Fe_3_S_4_ and PDS upon contact, likely facilitating an ETP‐induced decomposition of PDS, oxidation of iron active sites, and generation of ROS [30, 36]. Analogously, addition of CLO resulted in another significant current surge for FLO‐Fe_3_S_4_ catalytic systems, underscoring the effective electron transfer from CLO to FLO‐Fe_3_S_4_* complexes [44].

On the basis of these results, it can be reasonably inferred that CLO degradation in the FLO‐Fe_3_S_4_/PDS and OCT‐Fe_3_S_4_/PDS systems proceeded via SO_4_^•−^, •OH, O_2_^•−^, ^1^O_2_, ≡Fe(IV) = O, and ETP oxidation pathways, realizing an overall increase in the oxidizing capacity.

#### 2.3.2 Identification of Active Sites

**FIGURE 3:**
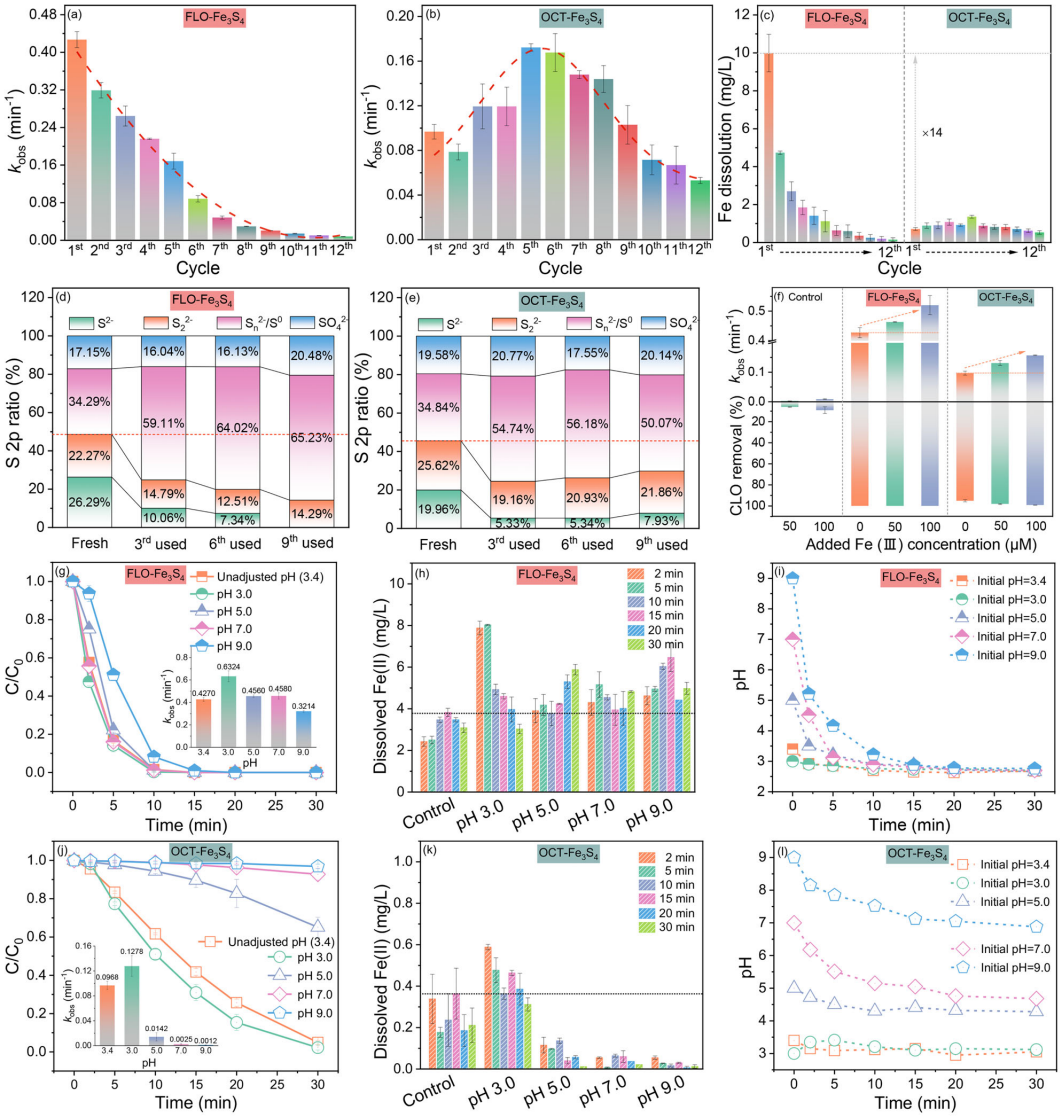
(a,b) Degradation rate of CLO, (c) leaching total Fe concentrations, and (d,e) relative content distributions of S species in catalysts in the FLO‐Fe_3_S_4_/PDS and OCT‐Fe_3_S_4_/PDS systems over 12 successive cycles. (f) Effects of exogenous Fe(III) ion addition on CLO degradation in the FLO‐Fe_3_S_4_/PDS and OCT‐Fe_3_S_4_/PDS systems. (g, j) The CLO degradation efficiencies, (h, k) leaching Fe(II) concentrations, and (i, l) variations of solution pH in the FLO‐Fe_3_S_4_/PDS and OCT‐Fe_3_S_4_/PDS systems under various pH conditions. Reaction conditions: [Catalysts] = 0.1 g/L, [PDS] = 1.0 mM, initial pH 3.40 (a–c), [CLO] = 5 mg/L, and T = 25°C.

Generally, cyclic stability is another important metric for catalysts. As depicted in Figure 3a and Figure S19a, the recyclability of FLO‐Fe_3_S_4_ progressively deteriorated, and only 22% of CLO was removed in the 12^th^ successive cycle with the *k*_obs_ value of 0.008 min^−1^. On the contrary, OCT‐Fe_3_S_4_ demonstrated exceptional reactivity for sustainably activating PDS across 12 cyclic experiments, with some even exhibiting a higher CLO degradation rate than the first cycle (Figure 3b and Figure S19b). More impressively, similar variation tendencies were observed in pre‐mixing experiments, as shown in Figure 2h, mixing PDS and FLO‐Fe_3_S_4_ in advance drastically interfered with CLO degradation, and such inhibitory effect was significantly enhanced with an extended premixing time. However, completely contrary results were observed for the OCT‐Fe_3_S_4_/PDS system, wherein the catalyst was combined with PDS prior to CLO addition revealed substantial improvement on CLO degradation rate (Figure 2i). In terms of metal ion leakage, the maximum concentration of total dissolved Fe was detected at 10 mg/L over a 30‐min reaction in the FLO‐Fe_3_S_4_/PDS system within 12 cyclic experiments, substantially higher than the 0.7 mg/L achieved by the OCT‐Fe_3_S_4_/PDS system (Figure 3c). The PDS utilization efficiency in the OCT‐Fe_3_S_4_/PDS system consistently stabilized after twelve cyclic reactions, which makes them suitable for repeated use in water treatment applications (Figure S20). But for FLO‐Fe_3_S_4_/PDS system, PDS decomposition gradually decreased, possibly because massive Fe species detached from catalyst surface as the reaction progresses, causing the deactivation of FLO‐Fe_3_S_4_.

Such interesting findings encourage us to conjecture that Fe sites may act as the dominant active sites, and the distinct PDS activation mechanisms by FLO‐Fe_3_S_4_ and OCT‐Fe_3_S_4_ might be attributed to different contents of Fe dissolution. To validate this hypothesis, oxalate and potassium thiocyanate (KSCN) were selected as the shielding reagents in further experiments, since they can strongly coordinate with Fe sites dispersed in the catalyst surface [45, 46]. For two reaction systems, introduction of oxalate and KSCN almost significantly suppressed CLO degradation (Figure S21), and similar inhibitory effects were also observed in premixing and oxalate synergistic influence experiments (Figure S22), collectively signifying that Fe sites served as the predominant contributing factors responsible for PDS activation [47]. Such a conclusion could also be corroborated by XPS analysis. In the Fe 2p spectra, the ratio of Fe(II)/Fe(III) in FLO‐Fe_3_S_4_ significantly decreased from 1.71 (fresh catalyst) to 0.90 (third used catalyst) but increased to 1.26 (sixth used catalyst) and 1.31 (ninth used catalyst) (Figure S23a). A similar variation tendency was also observed for the OCT‐Fe_3_S_4_ (Figure S23b), collectively reflecting the dynamic redox cycle of Fe(III)/Fe(II) during reaction. The FTIR spectra also revealed significant changes in vibrational peaks associated with Fe−S bond peaks at 615 cm^−1^ after reaction in two Fe_3_S_4_/PDS systems, indicative of the involvement of Fe−S groups (Figure S24) [48]. As for the XPS spectra of S 2p, the proportions of reductive S species (such as S^2−^ and S_2_^2−^) significantly reduced after PDS activation in two Fe_3_S_4_‐based catalytic systems, while the contents of S_n_^2−^/S^0^ and SO_4_^2−^ increased (Figure 3d,e). It is necessary to mention that the S^2−^ species could not directly activate PDS for the degradation of CLO (Figure S25). Therefore, S^2−^ species were likely to act as electron donors and provided electrons to facilitate the reduction of Fe (III) to Fe (II), followed by subsequent oxidation to the intermediate valence (S_2_^2−^ and S_n_^2−^/S^0^) and final sulfate species (SO_4_^2−^) (Equations (1), (2), (3)). Notably, the oxidation of S^2−^ to SO_4_^2−^ by Fe(III) did not occur in one step but involved the formation of intermediate sulfur species (S_2_^2−^/S_n_^2−^/S^0^). Once S^2−^ was exhausted, the cycling of Fe (III)/Fe (II) would be inhibited due to the non‐renewability of S^2−^, thereby weakening the activity of Fe_3_S_4_ catalyst [49]. The oxalate and KSCN poisoning experiments further indicated that S species predominantly served as electron donors for Fe(II) regeneration rather than PDS activation, because CLO degradation was completely inhibited after adding excess shielding scavengers. Overall, these findings compellingly demonstrated that the reductive S sites on Fe_3_S_4_ catalysts ensure the redox cycle between S^2−^/S^0^ and Fe(III)/Fe(II), and the regenerated Fe(II) species are conducive to sustaining catalytic activity [28].

To substantiate that the S species on Fe_3_S_4_ catalyst can facilitate Fe(II) regeneration during reaction, different amounts of exogenous Fe(III) ions were introduced into Fe_3_S_4_/PDS/CLO systems [45]. It can be clearly observed from Figure 3f that the degradation rate of CLO dramatically increased by 21.8% and 62.5% after adding 100 µM Fe(III) into the FLO‐Fe_3_S_4_/PDS and OCT‐Fe_3_S_4_/PDS systems, respectively; while negligible CLO removal was observed in the homogeneous Fe(III)/PDS system. Such results suggest that the exogenous Fe(III) synchronously interacts with the endogenous S of Fe_3_S_4_ catalyst, in which the S species on Fe_3_S_4_ surface will deliver electrons to the exogenous Fe(III) for Fe(II) regeneration, showing unique advantages in promoting Fe(III)/Fe(II) circulation during PDS activation [50].

Additionally, it has been reported that other non‐metallic sites like SVs can serve as the active sites, and the role of SVs in the reaction can be determined by Ag^+^ ion exchange strategy according to a previous study [25, 51]. As depicted in Figure S26, the CLO degradation rates were greatly decreased after removing the unsaturated S atoms of Fe_3_S_4_ crystals. The decrease in SVs concentrations in used Fe_3_S_4_ crystals also revealed the involvement of Svs in PDS activation and ROS generation (Figure S27), while FLO‐Fe_3_S_4_ crystal with higher SVs content exhibits higher catalytic activity (Figure S1).

(1) $$S^{2-}+Fe\left(III\right)\to S_{2}^{2-}+Fe\left(II\right)$$

(2) $$S_{2}^{2-}+Fe\left(III\right)\to S_{n}^{2-}+Fe\left(II\right)$$

(3) $$S_{n}^{2-}+Fe\left(III\right)\to S^{0}+Fe\left(II\right)$$

#### 2.3.3 Effect of Fe Dissolution on Catalytic Reaction

**FIGURE 4:**
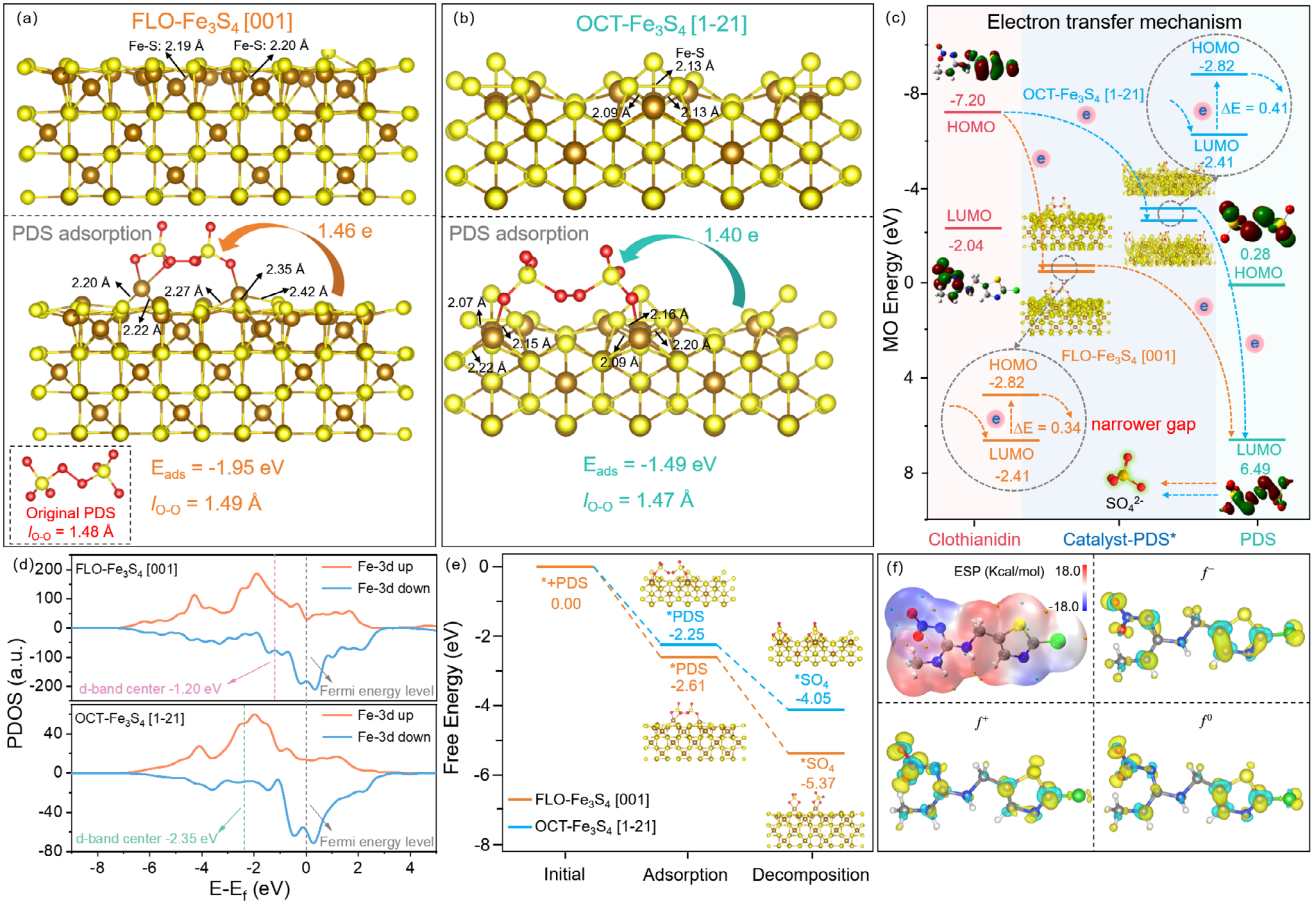
Adsorption energy and Bader charge of PDS adsorbed on (a) FLO‐Fe_3_S_4_ and (b) OCT‐Fe_3_S_4_. (c) Molecular orbital energies of CLO, FLO‐Fe_3_S_4_/PDS, OCT‐Fe_3_S_4_/PDS, and free PDS. (d) Projected density of states of Fe 3d orbitals in FLO‐Fe_3_S_4_ and OCT‐Fe_3_S_4_. (e) Gibbs free energies of *SO_4_ formation for FLO‐Fe_3_S_4_ and OCT‐Fe_3_S_4_ by PDS activation (inset: corresponding optimized intermediate configurations). (f) ESP distribution and equivalent surface images of ƒ^−^, ƒ^+^, and ƒ^0^ of CLO.

During the PDS activation process, the amount of Fe(II) is a key factor dominating the rate‐determining step, and the faster regeneration rate of Fe(II) endows the catalyst with higher catalytic performance [45]. As displayed in Figure S28, the detectable concentration of dissolved Fe(II) in the FLO‐Fe_3_S_4_/PDS system rapidly increased at 2 min and then fluctuated in equilibrium at a higher level (∼4 mg/L) than that in the OCT‐Fe_3_S_4_/PDS system (∼0.5 mg/L), which indicated that FLO‐Fe_3_S_4_ is more beneficial for the release of Fe species during reaction, possibly attributing to its longer Fe─S bonds on exposed [001] facet (Figure 4a,b). When 1,10‐phenanthroline was used as a shielding agent for Fe(II), CLO degradation was completely restricted, and a pink colored suspension was observed (Figure S29), further supporting that Fe(II) was continuously regenerated. Such results suggest that the PDS activation mechanism through Fe_3_S_4_ can be considered as homogeneous and heterogeneous processes, in which the homogeneous process refers to the continuous release of dissolved Fe(II) by Fe_3_S_4_ for PDS activation. For further validation, a comparative experiment of homogeneous and heterogeneous PDS activation systems was conducted, with results presented in Figure S30a. The homogeneous PDS activation systems constructed by filtrates obtained through separating FLO‐Fe_3_S_4_ and OCT‐Fe_3_S_4_ catalysts contributed to 43% and 5% of CLO degradation, respectively. Further introducing 0.5 mg/L and 4 mg/L of Fe(II) into the PDS/CLO systems, 8% and 33% of CLO could be degraded, respectively (Figure S30b). These results strongly indicate that the contribution of homogeneous PDS activation by leached Fe ions to CLO degradation in the FLO‐Fe_3_S_4_/PDS system was much greater than that in the OCT‐Fe_3_S_4_/PDS system. Therefore, it can be reasonably inferred that the decreased catalytic activity of FLO‐Fe_3_S_4_ in pre‐mixing and cyclic experiments (Figures 2h and 3a) can be attributed to the loss of Fe sites. Specifically, a large amount of Fe ions leaching from FLO‐Fe_3_S_4_ can induce homogeneous PDS activation for rapid ROS generation even under the absence of CLO, and these short‐lived radicals are constantly quenched during the pre‐mixing process, thereby impeding the degradation of CLO. In the other hand, massive Fe dissolution can accelerate the structural destruction of FLO‐Fe_3_S_4_. As displayed in Figure S31, SEM images demonstrated significant morphological alterations to FLO‐Fe_3_S_4_ after repeated reactions, such as partial pore blockage or structural reorganization; but no significant differences were observed in the microstructure of OCT‐Fe_3_S_4_ catalyst after reaction, indicating its exceptional structural integrity. These findings further confirm the challenge of balancing initial high catalytic activity with long‐term catalyst stability during heterogeneous catalytic reaction [52]. Gratifyingly, owing to the existence of reductive S species, the catalytic activity of OCT‐Fe_3_S_4_ evidently increased after introducing exogenous Fe(III) ions (Figure 3f), which overcomes the reactivity‐stability challenge to a certain extent, showing promise for practical application.

More interestingly, FLO‐Fe_3_S_4_ demonstrated an outstanding pH adaptability and sustained high catalytic activity (100% CLO removal within 30 min) across a wide initial pH range of 3.0–9.0 (Figure 3g), but OCT‐Fe_3_S_4_ showed diminished activities towards CLO degradation with increasing solution pH (Figure 3j). The point of zero charges (pHpzc) of two Fe_3_S_4_ crystals (Figure S32) were calculated as ∼7.0, indicating their similar adsorption capacities for negatively charged compounds during reaction [53]. To gain a more intuitive understanding of these differences, real‐time monitoring of Fe dissolution concentrations and pH variations were conducted in these systems. As shown in Figure 3h and Figure S33a, higher dissolved Fe(II) (∼4 mg/L) and total Fe (∼15 mg/L) were detected in the FLO‐Fe_3_S_4_/PDS system across the pH of 3.0–9.0 after 30 min reaction, but Fe dissolution was greatly decreased with increasing solution pH in the OCT‐Fe_3_S_4_/PDS systems (Figure 3k and Figure S33b). It can be reasonably inferred that massive Fe dissolution can induce homogeneous PDS activation in the FLO‐Fe_3_S_4_/PDS system under pH 3.0–9.0, generating more ROS for CLO degradation. Besides, it can be clearly observed that the addition of FLO‐Fe_3_S_4_ significantly decreased the final solution pH to approach 3.0 irrespective of the initial neutral or alkaline conditions (Figure 3i), unlike the OCT‐Fe_3_S_4_/PDS system (Figure 3l). The adjusted acidic conditions also ensured the activity of dissolved Fe ions, thereby promoting the homogeneous reaction.

To summarize, higher Fe(II) leached from FLO‐Fe_3_S_4_ ensures the rapid circulation of Fe(II)/Fe(III), which guarantees the subsequent occurrence of a series of more intensive homogeneous PDS activation reactions and ROS generation, thereby endowing FLO‐Fe_3_S_4_ with higher catalytic activity [25].

### 2.4 Theoretical Calculations

To delve into why the FLO‐Fe_3_S_4_ crystal with exposed [001] facet exhibits higher PDS activation capability than the OCT‐Fe_3_S_4_ with exposed [1‐21] facet, we employed density functional theory (DFT) calculations to explore their intrinsic facet‐activity relations. The optimized surface atomic configurations of the [001] and [1‐21] facets on Fe_3_S_4_ unit cell are presented in Figure 4a,b. The detailed electronic structures of the two exposed facets were illustrated by projected density of states (PDOS) [54, 55]. Analysis of the d‐band center (εd) of Fe 3d orbitals reveals that for the [001] facet, εd is located at −1.20 eV, which is closer to the Fermi level compared to the [1‐21] facet (Figure 4d). According to d‐band theory, a εd closer to the Fermi level suggests a stronger binding strength between catalytic sites and adsorbates, which is beneficial for transferring electrons from the catalyst to PDS, thereby promoting ROS generation [14, 56]. The adsorption energy (E_ads_) of [001] facet for PDS is calculated as −1.95 eV (Figure 4a), with its absolute value greatly exceeding that of the [1‐21] facet (−1.49 eV) (Figure 4b), strongly indicating that the [001] facet exhibits a stronger tendency to spontaneously bind with PDS. Bader charge analysis indicates that PDS acquires more electrons from the [001] facet (1.46 e), collectively signifying that the [001] facet is more favorable for electron transfer from the catalyst to PDS, which facilitates the adsorption and subsequent activation of PDS [57]. Correspondingly, the O−O bond length of the adsorbed PDS on [001] facet is longer than that on the [1‐21] facet (1.49 Å vs.1.47 Å), indicating that the O−O bond of adsorbed PDS on the [001] facet is more prone to breakage (Figure S9). Note that, the [001] facet on FLO‐Fe_3_S_4_ exhibits longer Fe−S bond lengths than that of the [1‐21] facet on OCT‐Fe_3_S_4_, even after combining with PDS (Figure 4a,b), which implies that the Fe−S bond is more likely to break in the FLO‐Fe_3_S_4_, thereby releasing more Fe ions for PDS activation (Figure 3c) [6, 58]. These results matched well with the above conclusion obtained: the superior PDS activation capability of FLO‐Fe_3_S_4_ can be ascribed to the simultaneous generation of abundant Fe(II), which induced a more intensive homogeneous reaction.

The molecular orbital (MO) energy levels of reactants were further conducted to unravel the inherent electron transfer mechanism over two catalytic systems. Generally, the highest occupied molecular orbital (HOMO) and the lowest unoccupied molecular orbital (LUMO) are attributed to the electron donor and acceptor orbital, respectively [59]. From Figure 4c, the HOMO of CLO (−7.20 eV) is more negative than the LUMO of FLO‐Fe_3_S_4_ [001]‐PDS* (−0.38 eV), denoting that the electrons can energetically favor the migration from HOMO (CLO) to LUMO (FLO‐Fe_3_S_4_ [001]‐PDS*). Then, the electrons localized in the HOMO of FLO‐Fe_3_S_4_ [001]‐PDS* (−0.72 eV) are readily transferred to the LUMO of PDS (6.55 eV), inducing an electrophilic attack on the O─O bond of PDS. The electron transfer from CLO to PDS via OCT‐Fe_3_S_4_ [1‐21]‐PDS* is also energetically favorable. Notably, the energy gap of FLO‐Fe_3_S_4_ [001]‐PDS* complex (0.34 eV) is narrower than that of OCT‐Fe_3_S_4_ [1‐21]‐PDS* (0.41 eV), illustrating that the electrons at FLO‐Fe_3_S_4_ [001]‐PDS* are more prone to transfer from the LUMO to HOMO, aligning with the electrochemistry analysis results (Figure 2f,g) [59].

The Gibbs free energy (ΔG) diagram and the corresponding optimized structures of intermediates are calculated in Figure 4e. Initially, PDS molecule thermodynamically adsorbed onto the surface of FLO‐Fe_3_S_4_ [001] and coordinated with Fe sites to form the transition state FLO‐Fe_3_S_4_ [001]‐PDS* complex, with an energy reduction of −2.61 eV. Then, the O─O bond in FLO‐Fe_3_S_4_ [001]‐PDS* was broken to spontaneously generate ROS and SO_4_*, reducing the Gibbs free energy to −5.37 eV. Compared with the [1‐21] facet of OCT‐Fe_3_S_4_ crystal (ΔG = −4.05 eV), PDS activation by the [001] facet of FLO‐Fe_3_S_4_ is more thermodynamically favorable for the generation of ROS, indicating that the exposed [001] facet confers a higher chemical reactivity and facilitates the electron transfer from FLO‐Fe_3_S_4_ to PDS [60].

**FIGURE 5:**
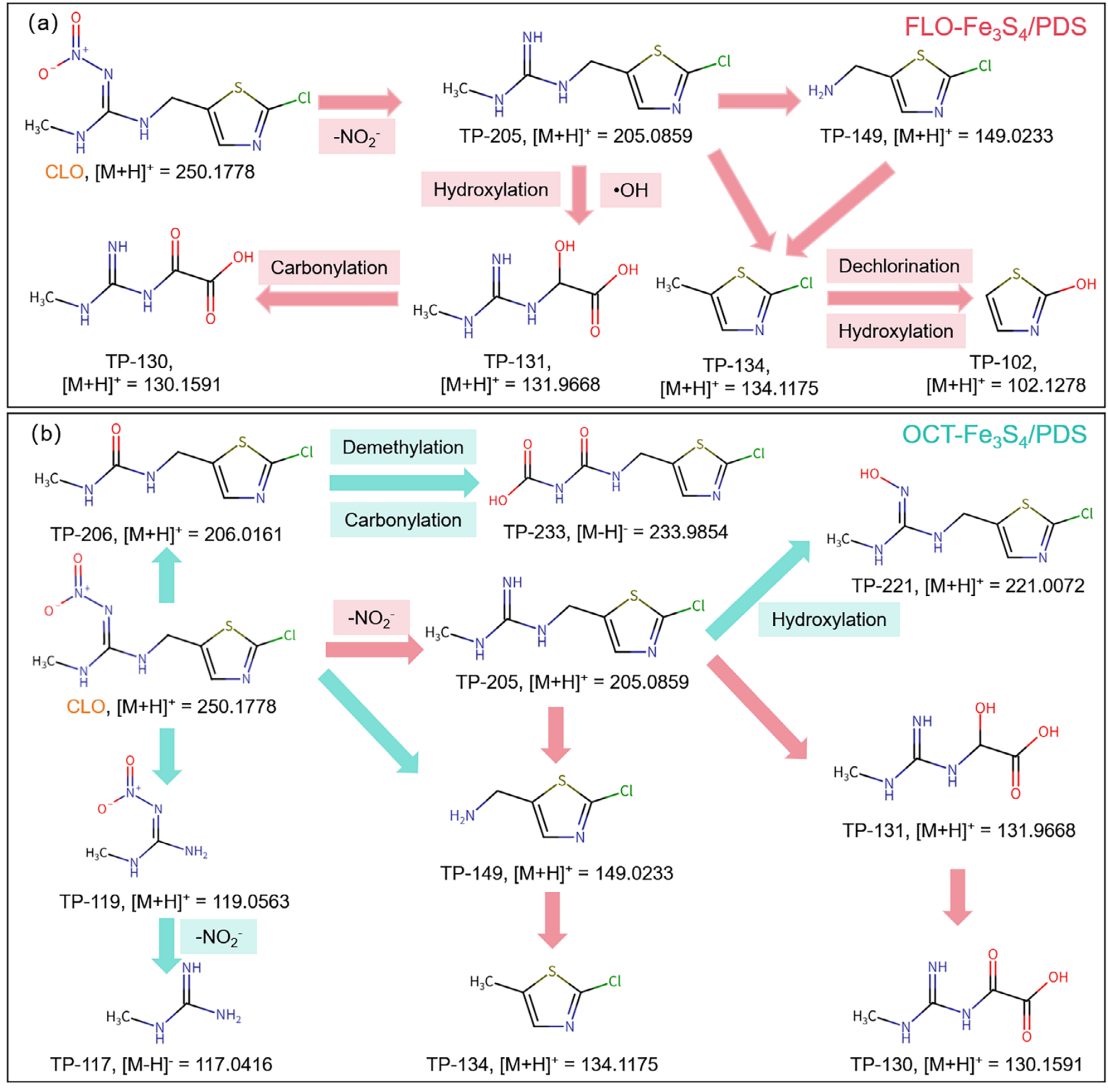
Possible degradation pathways of CLO in the (a) FLO‐Fe_3_S_4_/PDS and (b) OCT‐Fe_3_S_4_/PDS systems.

To better clarify the CLO degradation mechanism, the most vulnerable sites of CLO molecule were calculated (Figure S34). From the distribution of the electrostatic potential (ESP) electron cloud for CLO molecule (Figure 4f), it is observed that the electron‐rich region of CLO is mainly located on the nitroguanidine group, especially the ─N_2_O_2_ group, implying that this group is more vulnerable to attack [61]. The Fukui function (ƒ^+^, ƒ^−^ and ƒ^0^) (Figure 4f and Figure S35) also points to a similar conclusion, in which the C3, S5, Cl7, O16 and O17 sites exhibit relatively high ƒ^−^ values, suggesting that these sites are more susceptible to be attacked by electrophilic species such as ^1^O_2_ and ≡Fe(IV) = O [62]. Subsequently, ultra‐performance liquid chromatography‐quadrupole time‐of‐flight mass spectrometry (UPLC‐QTOF‐MS) was conducted to identify transformation products (TPs) in CLO degradation formed by FLO‐Fe_3_S_4_/PDS and OCT‐Fe_3_S_4_/PDS systems (Table S2). In the FLO‐Fe_3_S_4_/PDS system (Figure 5a), the intermediate product TP‐205 was identified as resulting from the electrophilic attack of the N–N bond of CLO, followed by the subsequent cleavage of C–N, demethylation, dechlorination, and hydroxylation reactions to form TP‐149, TP‐134, and TP‐102 [63]. Alternatively, TP‐205 could be attacked by •OH to yield TP‐131 and TP‐130 via electrophilic addition, hydroxylation, and carbonylation reactions [64]. In the OCT‐Fe_3_S_4_/PDS system (Figure 5b), in addition to the above degradation pathways, CLO could decompose to TP‐119 and TP‐117 via the cleavage of C─N and N─N bond. Product TP‐205 could transform to TP‐221 through the replacement of nitro group with a hydroxyl group. The direct oxidation of the –N_2_O_2_ group on CLO due to the electrophilic attack of N site caused the formation of TP‐206, which further transformed to TP‐233 via demethylation and carboxylation [63]. To sum up, denitration, hydroxylation, and branched chain cleavage are considered the major detoxification pathways for CLO in two systems.

### 2.5 Reaction Mechanisms

**FIGURE 6:**
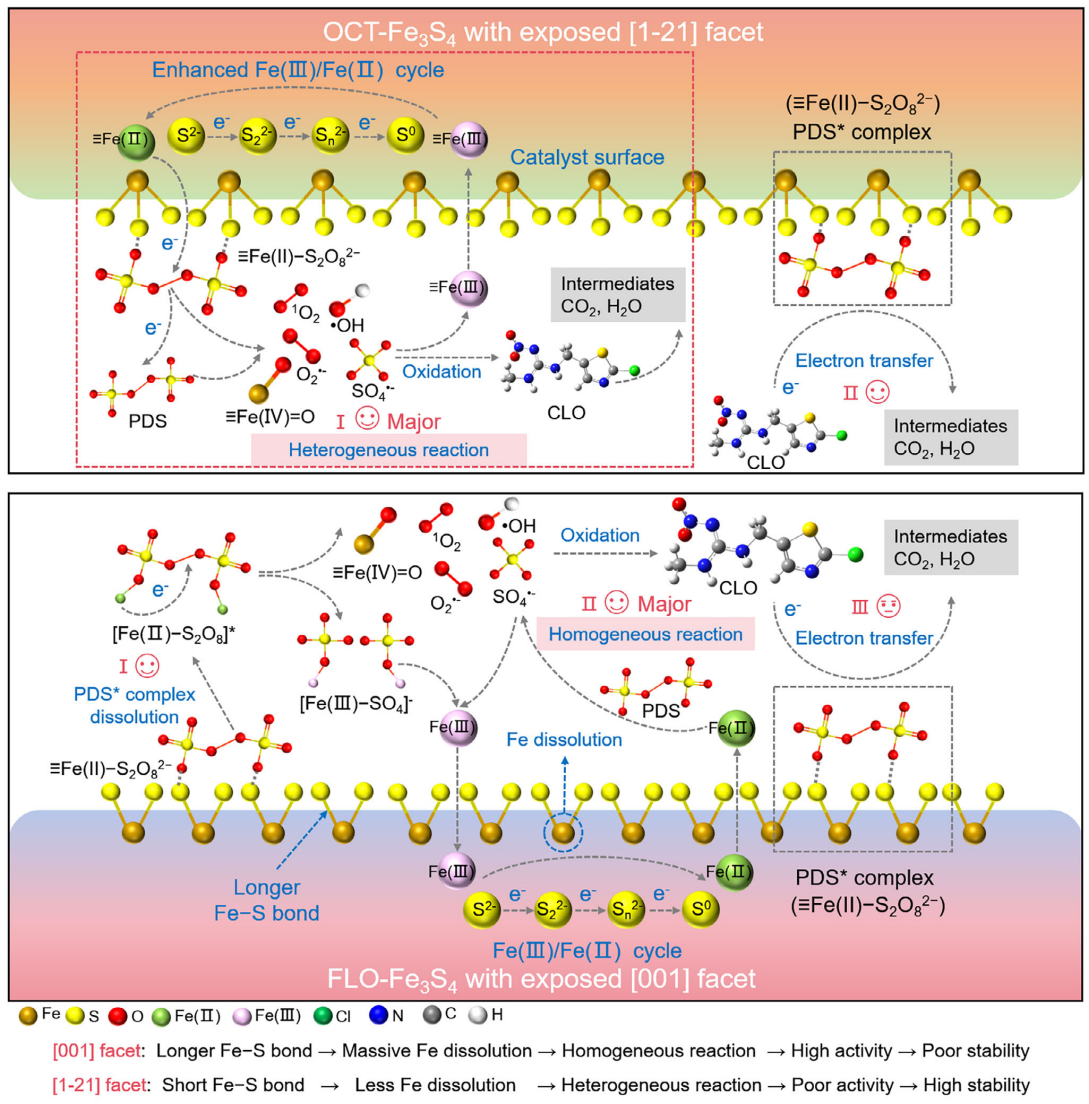
Reaction mechanisms of the FLO‐Fe_3_S_4_/PDS and OCT‐Fe_3_S_4_ systems.

Based on the above analyses and previous study [65], reasonable PDS activation mechanisms over FLO‐Fe_3_S_4_ and OCT‐Fe_3_S_4_ were proposed in Figure 6. First, the S_2_O_8_^2−^ groups preferentially adsorb on the surface of Fe_3_S_4_ catalysts and form ≡Fe(II)−S_2_O_8_^2−^complexes in two reaction systems. But for FLO‐Fe_3_S_4_, due to its longer Fe−S bonds, the formed ≡Fe(II)−S_2_O_8_^2−^ complex will detach from the FLO‐Fe_3_S_4_ surface into solution, and the dissociative Fe(II)−S_2_O_8_^2−^ complex can activate PDS to produce ROS for CLO degradation, accompanied by the generation of Fe(III) species. In a meanwhile, Fe species are more prone to release from FLO‐Fe_3_S_4_ surface, subsequently inducing a homogeneous activation process. After PDS activation, Fe(III) can accept electrons from the reductive S species in FLO‐Fe_3_S_4_ and is reduced to Fe(II), completing the cycling of Fe(III)/Fe(II). Regarding the OCT‐Fe_3_S_4_/PDS system, major Fe sites can be effectively preserved on the catalyst surface, which is more conducive to combining with PDS for the generation of ≡Fe(II)−S_2_O_8_^2−^complexes during reaction, ensuring that the heterogeneous activation process can be continued, unlike the FLO‐Fe_3_S_4_/PDS system. Thus, the increased catalytic activity of OCT‐Fe_3_S_4_ in pre‐mixing and cyclic experiments (Figures 2i and 3b) can be attributed to the accumulation of ≡Fe(II)−S_2_O_8_^2−^ complexes, which will facilitate the subsequent ETP‐mediated nonradical oxidation processes.

In summary, CLO degradation is dominated by two different mechanisms in this work: FLO‐Fe_3_S_4_‐controlled homogeneous PDS activation and OCT‐Fe_3_S_4_‐dominated heterogeneous PDS activation.

## 3 Conclusions

In recent years, Fe_3_S_4_ crystals have been regarded as a kind of promising catalysts for activating PDS to degrade organic pollutants in wastewater, but the intrinsic relationship between exposed facet and activity is still unclear. Hence, two distinct Fe_3_S_4_ crystals were successfully prepared and used to activate PDS for CLO degradation in this work. Mechanistic studies reveal that FLO‐Fe_3_S_4_ with preferentially exposed [001] facet and flower‐like structure exhibits higher reactivity for PDS activation to completely degrade CLO within 15 min, with a reaction rate of 0.4270 min^−1^, which is 4.4 times higher than that of the OCT‐Fe_3_S_4_ with exposed [1‐21] facet and octahedral structure. The superior activity of FLO‐Fe_3_S_4_ can be attributed to its longer Fe−S bonds on [001] facet, making them more prone to breaking and releasing more Fe ions for boosting homogeneous PDS activation. While the shorter Fe−S bond of OCT‐Fe_3_S_4_ alleviates Fe dissolution, thereby enhancing its catalytic stability and heterogeneous catalytic reaction. The leakage of Fe ions was detected at approximately 10 mg/L in the FLO‐Fe_3_S_4_/PDS system, significantly higher than that of the OCT‐Fe_3_S_4_/PDS system (0.7 mg/L). Quenching experiments, EPR spectra, and electrochemical tests collectively suggest that both of radical (SO_4_^•−^, •OH, O_2_^•–^, and ^1^O_2_) and nonradical (≡Fe(IV) = O and ETP) oxidation pathways are involved in CLO degradation in the two Fe_3_S_4_ catalytic systems. Further DFT calculations indicate that the [001] facet of FLO‐Fe_3_S_4_ can strengthen PDS adsorption (E_ads_: −1.95 eV vs. −1.49 eV) and enhance electron transfer (Q: 1.46 e vs. 1.40 e) compared with the [1‐21] facet of OCT‐Fe_3_S_4_, confirming the superiority of [001] over [1‐21] facet. The analyses of Fukui index and UPLC−QTOF−MS reveal the CLO degradation mechanism that the nitroguanidine group in CLO, especially the ─N_2_O_2_ group, is easy to be attacked by ROS. In summary, our study not only comprehensively elucidates the PDS activation mechanisms over Fe_3_S_4_ crystals with distinct exposed facets and establishes a facet−activity relationship chain of “facet/morphology → Fe−S length → Fe dissolution → catalytic activity”, but also unravels the overlooked effect of Fe−S length on catalytic reaction, providing vital enlightenment on the enhanced catalytic activity by regulating the exposed facet of the catalyst.

## 4 Experimental Section

Details of reagents, characterization of Fe_3_S_4_ crystals, analytical methods, and theoretical calculations are provided in Supporting Information (SI).

### 4.1 Synthesis of Catalysts

Fe_3_S_4_ crystals with distinct morphologies and facets were synthesized via one‐step hydrothermal strategies. In a typical synthesis of flower‐like Fe_3_S_4_ (denoted as FLO‐Fe_3_S_4_) [66], FeCl_3_·6H_2_O (3.0 mmol) and thiourea (6.0 mmol) were mixed in 60 mL ethylene glycol and continuously stirred until the mixture was clear. The homogeneous solution was transferred to a 75 mL Teflon‐lined stainless‐steel autoclave and hydrothermally reacted at 180°C for 12 h. After naturally cooling down to room temperature, the black powder was collected and washed with distilled water and ethanol several times, subsequently freeze‐dried under vacuum conditions overnight. For the preparation of octahedral Fe_3_S_4_ (denoted as OCT‐Fe_3_S_4_) [10], 0.6 mmol cetyltrimethyl ammonium bromide (CTAB) was dissolved in 35 mL of deoxygenated water, then 3 mmol L‐cysteine and 2 mmol FeCl_2_·4H_2_O were added to the above solution and stirred for 10 min. Subsequently, the solution was transferred to a 75 mL Teflon‐sealed autoclave and heated at 165°C for 40 h.

### 4.2 Experimental Procedures

A catalytic reaction was initiated in a 50 mL glass vial containing 40 mL mixture (5 mg/L CLO, 1.0 mM PDS, initial pH 3.40). The reaction was triggered by introducing 4 mg of Fe_3_S_4_ and shaken on a rotary shaker (BSD‐YX2400, Boxun, China) at 150 rpm and 25°C. After predetermined time intervals, 0.7 mL of liquid samples were filtered through 0.22 µm nylon membranes into vials preloaded with 0.7 mL of MeOH for further analysis. Sulfuric acid and sodium hydroxide were used to adjust the initial pH of the reaction solution if needed. All catalytic degradation experiments were independently conducted in triplicate (n = 3) unless otherwise specified, and the data are presented as mean ± standard deviation (SD).

A pseudo first‐order kinetic model was used to simulate the kinetics for degradation of the contaminants in this study, and the degradation rate constant k_obs_ was calculated by:

(4) $$-\ln\left(C_{t}/C_{0}\right)=k_{\mathrm{obs}}\cdot t$$

In this equation, C_0_ is the initial concentration of the contaminant, and C_t_ is the concentration of the contaminant at time t. Thus, k_obs_ can be graphically determined by plotting ‐ln (C_t_/C_0_) vs. t.

## Author Contributions

**Jiaqu Tan**: Writing – original draft, investigation, formal analysis, data curation. **Min Yu**: Investigation. **Jinfeng Wang**: Methodology. **Dongya Li**: Software. **Fan Yang**: Software. **Yulong Zhang**: Resources. **Xueming Lin**: Writing – review & editing, supervision, funding acquisition.

## Conflicts of Interest

The authors declare no conflicts of interest.

## Supporting information




**Supporting File**: advs76644‐sup‐0001‐SuppMat.docx.

## Data Availability

The data that support the findings of this study are available in the supplementary material of this article.
