# Supplementary material for: Facet‐Dependent Activity of Fe3S4 Crystal in Peroxydisulfate Activation for Organic Pollutants Degradation
Source: Adv Sci (Weinh). 2026 Jul 17;13(56):e76644. doi: 10.1002/advs.76644 (PMC13379216; doi:10.1002/advs.76644)
Supplement: Supplementary file 1 [file ADVS-13-e76644-s001.docx]

**Supporting Information**

Facet-dependent Activity of Fe_3_S_4_ Crystal in Peroxydisulfate Activation for Organic Pollutants Degradation

Jiaqu Tan ^a, b, d^, Min Yu ^a, c^, Jinfeng Wang ^e^, Dongya Li ^f^, Fan Yang ^g^,

Yulong Zhang ^a, b, d^, and Xueming Lin ^a, c, *^

^a^ College of Natural Resources and Environment, South China Agricultural University, Guangzhou 510642, P. R. China

^b^ Guangdong Research Center for Agricultural Soil Pollution Prevention and Control Engineering Technology, College of Natural Resources and Environment, South China Agricultural University, Guangzhou 510642, P. R. China

^c^ Guangdong Provincial Key Laboratory of Agricultural & Rural Pollution Abatement and Environmental Safety, Guangzhou 510642, P. R. China

^d^ Key Laboratory of Arable Land Conservation (South China), MOA, College of Natural Resources and Environment, South China Agricultural University, Guangzhou 510642, P. R. China; Guangdong

^e^ State Key Laboratory of Pollution Control and Resource Reuse, School of the Environment, Nanjing University, Nanjing 210023, P. R. China.

^f^ School of Environmental Engineering, Wuhan Textile University, Wuhan 430073, P. R. China.

^g^ School of electronic and electrical engineering, Wuhan Textile University, Wuhan 430200, P. R. China

*Corresponding author: Xueming Lin

Email addresses: xm.lin@scau.edu.cn (X. Lin)

This supplementary material includes 55 Pages (including this page), 9 Texts, 35 Figures, and 3 Tables.

**CONTENTS**

**Texts**

**Text S1.** Chemicals and reagents

**Text S2.** Characterization of Fe_3_S_4_ crystals

**Text S3.** Theoretical calculations

**Text S4.** Measurement of PDS concentration

**Text S5.** Measurement of dissolved Fe(II) and dissolved total iron concentrations

**Text S6.** Evaluation of contribution ratios for ROS in CLO degradation

**Text S7.** Microwave digestion for ICP-OES analysis

**Text S8.** Electrochemical tests

**Text S9.** Detailed procedures of UPLC–QTOF–MS analysis

**Figures**

**Figure S1.** EPR spectra of FLO-Fe_3_S_4_ and OCT-Fe_3_S_4_.

**Figure S2.** Survey XPS spectra of FLO-Fe_3_S_4_ and OCT-Fe_3_S_4_.

**Figure S3.** ICP-OES analysis of FLO-Fe_3_S_4_ and OCT-Fe_3_S_4_.

**Figure S4.** Relative content distributions of S species in FLO-Fe_3_S_4_ and OCT-Fe_3_S_4_.

**Figure S5.** High resolution XPS spectra of O 1s for FLO-Fe_3_S_4_ and OCT-Fe_3_S_4_.

**Figure S6.** (a) The N_2_ adsorption-desorption isotherms and (b) the corresponding pore size distribution of FLO-Fe_3_S_4_ and OCT-Fe_3_S_4_.

**Figure S7.** VSM images of (a) FLO-Fe_3_S_4_ and (b) OCT-Fe_3_S_4_.

**Figure S8.** Adsorption of CLO in various reaction systems.

**Figure S9.** (a) The standard calibration curve for PDS measurement. (b) PDS decomposition efficiencies in the FLO-Fe_3_S_4_/PDS and OCT-Fe_3_S_4_/PDS systems.

**Figure S10.** A comparison between Fe_3_S_4_ and other heterogeneous metal catalysts in activating PDS to degrade contaminants.

**Figure S11.** Effects of (a) PDS concentration and (b) catalyst dosage on CLO degradation in the FLO-Fe_3_S_4_/PDS system.

**Figure S12.** Effect of reaction temperature on CLO degradation in the (a) FLO-Fe_3_S_4_/PDS and (b) OCT-Fe_3_S_4_/PDS systems. Calculation of activation energy for (c) FLO-Fe_3_S_4_ and (d) OCT-Fe_3_S_4_ by the plot of ln(k_obs_) against 1/T for a range of temperatures.

**Figure S13.** Effects of common anions (Cl^−^ and NO_3_^−^) and natural organic matter (NOM) on CLO degradation in the (a-c) FLO-Fe_3_S_4_/PDS and (d-f) OCT-Fe_3_S_4_/PDS systems.

**Figure S14.** Degradation efficiencies of different contaminants in the FLO-Fe_3_S_4_/PDS and OCT-Fe_3_S_4_/PDS systems.

**Figure S15.** Spin-trapping EPR signals for O_2_^•−^ in different Fe_3_S_4_/PDS systems.

**Figure S16.** The conversion (η) of PMSO_2_/PMSO in the FLO-Fe_3_S_4_/PDS and OCT-Fe_3_S_4_/PDS systems.

**Figure S17.** Effects of dissolved oxygen and D_2_O on CLO degradation in the FLO-Fe_3_S_4_/PDS and OCT-Fe_3_S_4_/PDS systems.

**Figure S18.** Calculated contributions from different ROSs to CLO degradation in the (a) FLO-Fe_3_S_4_/PDS and (b) OCT-Fe_3_S_4_/PDS systems.

**Figure S19.** Degradation efficiencies of CLO in the (a) FLO-Fe_3_S_4_/PDS and (b) OCT-Fe_3_S_4_/PDS systems over 12 successive cycles.

**Figure S20.** PDS decomposition efficiencies in the FLO-Fe_3_S_4_/PDS and OCT-Fe_3_S_4_/PDS systems over 12 successive cycles.

**Figure S21.** Effects of oxalate and KSCN on CLO degradation in the (a, c) FLO-Fe_3_S_4_/PDS and (b, d) OCT-Fe_3_S_4_/PDS systems.

**Figure S22.** Synergistic effects of pre-mixing experiment and oxalate on the OCT-Fe_3_S_4_/PDS system.

**Figure S23.** High-resolution XPS spectra of Fe 2p for fresh and used Fe_3_S_4_ catalysts: (a) FLO-Fe_3_S_4_ and (b) OCT-Fe_3_S_4_.

**Figure S24.** FTIR spectra of fresh and used catalysts: (a) FLO-Fe_3_S_4_ and (b) OCT-Fe_3_S_4_.

**Figure S25.** Effect of S^2-^ ion on PDS activation for CLO degradation.

**Figure S26.** (a-b) Experiments of unsaturated S removed.

**Figure S27.** EPR spectra of fresh and used catalysts: (a) FLO-Fe_3_S_4_ and (b) OCT-Fe_3_S_4_.

**Figure S28.** The Fe(Ⅱ) leaching amount of different catalyst during PDS activation.

**Figure S29.** Scavenging effects of phenanthroline on CLO degradation in the FLO-Fe_3_S_4_ and OCT-Fe_3_S_4_/PDS systems.

**Figure S30.** (a) CLO degradation efficiencies via PDS activation by filtrates obtained through separating FLO-Fe_3_S_4_ and OCT-Fe_3_S_4_ catalysts. (b) Effect of Fe(Ⅱ) ion on PDS activation.

**Figure S31.** SEM images of fresh and used catalysts: (a-d) FLO-Fe_3_S_4_ and (e-h) OCT-Fe_3_S_4_.

**Figure S32.** Zeta potentials of FLO-Fe_3_S_4_ and OCT-Fe_3_S_4_ at different pH.

**Figure S33.** The total Fe leaching concentrations of (a) FLO-Fe_3_S_4_ and (b) OCT-Fe_3_S_4_ under different pH conditions.

**Figure S34.** The chemical structure of CLO.

**Figure S35.** Fukui index of CLO.

**Tables**

**Table S1.** Comparison of various catalysts in recently reported PDS activation processes.

**Table S2.** The identified intermediates of CLO by UPLC−QTOF−MS in the FLO-Fe_3_S_4_/PDS and OCT-Fe_3_S_4_/PDS systems. (a) Positive ion mode, (b) Negative ion mode.

**Table S3.** The HPLC analysis detection parameters for different pollutants.

**Supplementary Texts**

**Text S1. Chemicals and reagents**

Peroxydisulfate (NaS_2_O_8_, ≥99.0%, Damao, China), clothianidin (CLO, ≥95.0%, Aladdin, China), thiamethoxam (THM, ≥95.0%, Macklin, China), imidacloprid (IMI, ≥ 97.0%, Macklin, China), ferric chloride (FeCl_3_·6H_2_O, ≥ 99.0%, Macklin, China), thiourea (≥ 99.0%, Macklin, China), ethylene glycol (≥ 99.0%, Macklin, China), ferrous chloride (FeCl_2_.4H_2_O, ≥ 99.0%, Macklin, China), cetyl-trimethyl ammonium bromide (CTAB, ≥99.0%, Macklin, China), L-cysteine (≥99.0%, Aladdin, China), ferrous sulfate (FeSO_4_·7H_2_O, ≥99.0%, Macklin, China), ethanol (EtOH, ≥99.0%, Anpel, China), methanol (MeOH, ≥99.0%, Anpel, China), tert-butanol (TBA, ≥99.0%, Anpel, China), 1,10-phenanthroline (phen, ≥97.0%, Macklin, China), deuterium oxide (D_2_O, 99.9 atom% D, Macklin, China), sulfuric acid (H_2_SO_4_, ≥98.0%, Guangzhou Chemical, China), sodium hydroxide (NaOH, ≥99.0%, Guangzhou Chemical, China), sodium chloride (NaCl, ≥99.0%, Guangzhou Chemical, China), sodium nitrate (NaNO_3_, ≥99.0%, Guangzhou Chemical, China), sodium oxalate (≥99.9%, Macklin, China), ethylenediaminetetraacetic acid (EDTA, ≥99.9%, Macklin, China), dimethyl sulfoxide were used as received without additional purification. Standard Suwannee River Nature organic matter (SR-NOM), obtained from the International Humic Substances Society, was used as a model NOM to investigate the interference of possible coexisting NOM on pollutant degradation. Ultrapure water (Milli-Q, 18.2 MΩ cm) was produced from a purification system (Millipore, USA) and used as required in all experiments.

**Text S2. Characterization of Fe_3_S_4_ crystals**

The micromorphology and elemental composition of the prepared Fe_3_S_4_ crystal was characterized by scanning electron microscopy (SEM, Sigma 300, Zeiss, Germany) and transmission electron microscopy (TEM, FEI Tecnai G2 F20 S-Twin 200 kV, USA). X-ray diffraction (XRD, D8 advance, Bruker, Germany) with a Cu-Kα radiation source operating at 45 kV and 15 mA was used to analyze the crystal structure of the prepared samples. The functional groups of the fresh and used Fe_3_S_4_ crystals were measured by a Fourier transform infrared spectrometer (FTIR, Nicolet IS5, Thermo Fisher Scientific, USA). The chemical states of the Fe_3_S_4_ samples were analyzed by X-ray photoelectron spectroscopy (XPS, ESCALAB 250XI, Thermo Fisher Scientific, USA) equipped with a dual X-ray Al-Kα source. The magnetism of Fe_3_S_4_ was determined using a vibration sample magnetometer (VSM, LakeShore 7404, USA). EPR signals for reactive oxidative species (ROS) were obtained by using an electro paramagnetic resonance spectrometer (EPR, JES-FA300, JEOL, Japan), and 5,5-dimethyl-1-pyrroline-N-oxide (DMPO, ≥97%, Sigma–Aldrich, USA.) and 2,2,6,6-tetramethylpiperidine (TEMP, ≥98%, Sigma–Aldrich, USA) were used as spin-trapping agents.

**Text S3. Theoretical calculations**

All first-principles calculations were performed using density functional theory (DFT) as implemented in the Vienna *Ab initio* Simulation Package (VASP) [1]. The electron–ion interactions were described by the projector augmented-wave (PAW) method [2], and the exchange–correlation energy was treated within the generalized gradient approximation (GGA) using the Perdew–Burke–Ernzerhof (PBE) functional [3]. The plane-wave basis set was expanded with a kinetic energy cutoff of 450 eV. Electronic occupancies were treated using the first-order Methfessel–Paxton smearing with a smearing width of 0.2 eV [4]. Long-range dispersion interactions were accounted for by the Grimme DFT-D3 correction [5]. Spin-polarized calculations were considered with an initial magnetic moment of 3.0 μB assigned to each Fe atom.

Geometry optimizations were carried out until the total energy change between two consecutive ionic steps was below 1×10^−5^ eV and the residual forces on each atom were smaller than 0.02 eV/Å. The simulation slab model including [001] and [1-21] surfaces were constructed with vacuum spacings of at least 15 Å along z axis to eliminate spurious interactions between periodic images. A 2×2×1 Monkhorst–Pack grid was employed for structure optimization, the frequency calculation and the bader charge calculation, while a denser grid 4×4×1 was used for density of states calculation.

The Gibbs free energy G was calculated using the formula G = E + ZPE -TS, while E, ZPE and S represents the internal energy, the zero-point energy and entropy, respectively. The ZPE and S were estimated from the corresponding frequency calculations.

The nucleophilic and electrophilic attack on CLO molecular were performed using the Gaussian 16 software [6]. The B3LYP functional was adopted for all calculations in combination with the D3BJ dispersion correction [7-8]. For geometry optimization and frequency calculations, 6-31G(d,p) basis set was used for all atoms [9-10]. The single point calculations under the level of B3LYP-D3BJ/6-311+G(d,p) [11]. Fukui function [12] is performed in Multiwfn [13] package.

**Text S4. Measurement of PDS concentration**

The PDS concentration was quantified with a potassium iodide spectrophotometric method. More details are as follows:

1. Mixture: 0.4 g KI + 0.2 mL Sample + 0.02 g NaHCO_3_ + 4.8 mL Ultrapure water

2. Reaction time: 20 min

3. Detection λ: 352 nm

**Text S5. Measurement of dissolved Fe(II) and dissolved total iron concentrations**

The concentrations of dissolved Fe(II) and dissolved total iron in reaction solution was detected with a phenanthroline spectrophotometric method. After predetermined time intervals, 1 mL of solution was obtained by filtration. For the detection of Fe(II) ion, 0.4 mL of 1.5 g/L 1,10-phenanthroline and 1 mL of 1 M sodium acetate were added to the 1 mL filtered solution for mixing 10 min. Afterward, the solution was analyzed by UV-vis spectrometry at 510 nm. To measure total iron concentrations, 0.2 mL of 100 g/L hydroxylamine hydrochloride was added to the above solution to reduce Fe(III) to Fe(II). The solution was then analyzed at the same wavelength.

**Text S6. Evaluation of contribution ratios for ROS in CLO degradation**

The contribution ratios (CR) of different ROS were estimated using the approach provided by Hu et al. [14]. The rate constant without adding any scavengers was set as k_0_, and that with the addition of MeOH, TBA, p-BQ, FFA, and DMSO was set as k_MeOH_, k_TBA_, k_p-BQ_ kF_FA_, and k_DMSO_, respectively. The contribution of each reactive species to the degradation of CLO was determined using Eqs. (S1)~(S5).

CR(•OH) = (k_0_ - k_TBA_)/k_0_ × 100% (S1)

CR(SO_4_^•−^) = (k_TBA_ - k_MeOH_)/k_0_ × 100% (S2)

CR(^1^O_2_) = (k_0_ - k_FFA_)/k_0_ × 100% (S3)

CR(O_2_^•−^) = (k_0_ - k_p-BQ_)/k_0_ × 100% (S4)

CR(≡Fe(IV)=O) = (k_0_ - k_DMSO_)/k_0_ × 100% (S5)

**Text S7. Microwave digestion for ICP-OES analysis**

50 mg Fe_3_S_4_ crystal was placed into a digestion vessel, followed by the addition of 10 mL acid solution (HNO_3_: HF: HCl = 1:1:3). The sealed digestion vessel was then digested at 180°C for 90 minutes using a microwave digestion system. Following digestion, the vessel was opened and heated at 150 ^°^C for 2 hours to evaporate residual acids. After cooling to room temperature, the solution was diluted to a final volume of 50 mL for subsequent ICP-OES analysis of Fe ion concentrations.

**Text S8. Electrochemical tests**

All electrochemical experiments were performed on an electrochemical workstation (660E, CHI, China) with a conventional three-electrode system. A glassy carbon electrode loaded with catalyst was first prepared. 20 mg of Fe_3_S_4_ was dispersed into a mixture of 0.2 mL of Nafion® solution (5.0 wt%) and 2 mL of ethanol, and then sonicated for 30 min. Subsequently, 20 μL of suspension was dropped onto the surface of the glassy carbon electrode and dried at 60 ^°^C overnight. Pt and silver/silver chloride (Ag/AgCl) electrodes were used as a counter electrode and a reference electrode, respectively. Chronoamperometry was carried out at the bias of 0.0 V (vs. Ag/AgCl) with 50 mM Na_2_SO_4_ as supporting electrolyte. Electrochemical impedance spectroscopy (EIS) was measured at open potential with a scan frequency in the range from 10^5^ to 10^-1^ Hz.

**Text S9. Detailed procedures of UPLC–QTOF–MS analysis**

The aqueous phase of the reaction was extracted and concentrated with the LC-18 SPE column (6 mL/500 mg, Supelco, USA), which were conditioned by flushing with 6 mL methanol and 6 mL water. Then, 200 mL sample was loaded, and the analyte was eluted with 6 mL of methanol. The UPLC–QTOF–MS analysis was conducted under the following conditions: separation was performed with a ECLIPS PLUS C18 column (100 mm × 2.1 mm, 1.8 μm; Agilent, USA) and eluted with gradient solvent from 10% A: 90% B (0 ~ 2min) to 90% A: 10% B (2 ~ 11min) and 10% A: 90% B (11 ~ 15min) at a flow rate of 0.300 mL/min, where A is methanol and B is 0.2% (v/v) aqueous formic acid. The column was maintained at 40 °C. Accurate MS/MS patterns of THM and its degradation products were analyzed in a molecular ion scanning mode (m/z 50 to 1100) in negative and positive electrospray ionization (dual AJS ESI) modes. The molecular formulas of identified products were proposed on the basis of experimental and theoretical m/z values.

**Supplementary Figures**

**Figure S1.** EPR spectra of FLO-Fe_3_S_4_ and OCT-Fe_3_S_4_.

**Figure S2.** Survey XPS spectra of FLO-Fe_3_S_4_ and OCT-Fe_3_S_4_.

**Figure S3.** ICP-OES analysis of FLO-Fe_3_S_4_ and OCT-Fe_3_S_4_.

**Figure S4.** Relative content distributions of S species in FLO-Fe_3_S_4_ and OCT-Fe_3_S_4_.

**Figure S5.** High resolution XPS spectra of O 1s for FLO-Fe_3_S_4_ and OCT-Fe_3_S_4_.

**Figure S6.** (a) The N_2_ adsorption-desorption isotherms and (b) the corresponding pore size distribution of FLO-Fe_3_S_4_ and OCT-Fe_3_S_4_.

**Figure S7.** VSM images of (a) FLO-Fe_3_S_4_ and (b) OCT-Fe_3_S_4_.

**Figure S8.** Adsorption of CLO in various reaction systems. Reaction conditions: [Catalysts] = 0.1 g/L (a), [PDS] = 1.0 mM (b), unadjusted pH, [CLO] = 5 mg/L, and T = 25 ^◦^C.

**Figure S9.** (a) The standard calibration curve for PDS measurement. (b) PDS decomposition efficiencies in the FLO-Fe_3_S_4_/PDS and OCT-Fe_3_S_4_/PDS systems. Reaction conditions: [Catalysts] = 0.1 g/L, [PDS] = 1.0 mM, initial pH = 3.40, [CLO] = 5 mg/L, and T = 25 ^◦^C.

**Figure S10.** A comparison between Fe_3_S_4_ and other heterogeneous metal catalysts in activating PDS to degrade contaminants.

**Figure S11.** Effects of (a) PDS concentration and (b) catalyst dosage on CLO degradation in the FLO-Fe_3_S_4_/PDS system. Reaction conditions: [Catalysts] = 0.1 g/L (a), [PDS] = 1.0 mM (b), unadjusted pH, [CLO] = 5 mg/L, and T = 25 ^◦^C.

**Figure S12.** Effect of reaction temperature on CLO degradation in the (a) FLO-Fe_3_S_4_/PDS and (b) OCT-Fe_3_S_4_/PDS systems. Calculation of activation energy for (c) FLO-Fe_3_S_4_ and (d) OCT-Fe_3_S_4_ by the plot of ln(k_obs_) against 1/T for a range of temperatures. Reaction conditions: [Catalysts] = 0.1 g/L, [PDS] = 1.0 mM, unadjusted pH, [CLO] = 5 mg/L, and T = 25−45 ^◦^C.

Note: The activation energy (E_a_) was evaluated by determining the reaction rate constants at each temperature using first-order kinetics and the Arrhenius equation (Eq. S6):

$\text{Ln}\left( \text{k}_{\text{obs}} \right)\text{=-}\frac{\text{E}_{\text{a}}}{\text{RT}}\text{+}\text{LnA}$ (S6)

where k_obs_ represents the reaction rate constant that changes with temperature, A stands for the pre-exponential, Arrhenius, or frequency factor, E_a_ is the activation energy, T stands for the absolute temperature, and R is the universal gas constant (8.314 J/mol·k).

**Figure S13.** Effects of common anions (Cl^−^ and NO_3_^−^) and natural organic matter (NOM) on CLO degradation in the (a-c) FLO-Fe_3_S_4_/PDS and (d-f) OCT-Fe_3_S_4_/PDS systems. Reaction conditions: [Catalysts] = 0.1 g/L, [PDS] = 1.0 mM, unadjusted pH, [CLO] = 5 mg/L, and T = 25 ^◦^C.

**Figure S14.** Degradation efficiencies of different contaminants in the FLO-Fe_3_S_4_/PDS and OCT-Fe_3_S_4_/PDS systems. Reaction conditions: [Catalysts] = 0.1 g/L, [PDS] = 1.0 mM, initial pH = 3.40, [Contaminants] = 5 mg/L, and T = 25 ^◦^C.

**Figure S15.** Spin-trapping EPR signals for O_2_^•−^ in different Fe_3_S_4_/PDS systems.

**Figure S16.** The conversion (η) of PMSO_2_/PMSO in the FLO-Fe_3_S_4_/PDS and OCT-Fe_3_S_4_/PDS systems.

**Figure S17.** Effects of dissolved oxygen and D_2_O on CLO degradation in the FLO-Fe_3_S_4_/PDS and OCT-Fe_3_S_4_/PDS systems. Reaction conditions: [Catalysts] = 0.1 g/L, [PDS] = 1.0 mM, initial pH = 3.40, [CLO] = 5 mg/L, and T = 25 ^◦^C.

**Figure S18.** Calculated contributions from different ROSs to CLO degradation in the (a) FLO-Fe_3_S_4_/PDS and (b) OCT-Fe_3_S_4_/PDS systems.

**Figure S19.** Degradation efficiencies of CLO in the (a) FLO-Fe_3_S_4_/PDS and (b) OCT-Fe_3_S_4_/PDS systems over 12 successive cycles. Reaction conditions: [Catalysts] = 0.1 g/L, [PDS] = 1.0 mM, initial pH = 3.40, [CLO] = 5 mg/L, and T = 25 ^◦^C.

**Figure S20.** PDS decomposition efficiencies in the FLO-Fe_3_S_4_/PDS and OCT-Fe_3_S_4_/PDS systems over 12 successive cycles. Reaction conditions: [Catalysts] = 0.1 g/L, [PDS] = 1.0 mM, initial pH = 3.40, [CLO] = 5 mg/L, and T = 25 ^◦^C.

**Figure S21.** Effects of oxalate and KSCN on CLO degradation in the (a, c) FLO-Fe_3_S_4_/PDS and (b, d) OCT-Fe_3_S_4_/PDS systems. Reaction conditions: [Catalysts] = 0.1 g/L, [PDS] = 1.0 mM, initial pH = 3.40, [CLO] = 5 mg/L, and T = 25 ^◦^C.

**Figure S22.** Synergistic effects of pre-mixing experiment and oxalate on the OCT-Fe_3_S_4_/PDS system. (“*Pre-mixing 90 min + 10 mM oxalate*” means that oxalate was added after pre-mixing catalyst and PDS; “*10 mM oxalate + pre-mixing 90 min*” means that oxalate was first added and then pre-mixing catalyst, oxalate and PDS.)

**Note:** The pre-mixing experiment and oxalate synergistic influence experiment was further supplemented in this work. As shown in Figure S22, on the premise of premixing experiment, the introduction of oxalate could greatly inhibit the degradation of CLO in the OCT-Fe_3_S_4_/PDS system, suggesting that when Fe sites in Fe_3_S_4_ were shielded by oxalate, the Fe_3_S_4_-PDS* complexes-induced ETP oxidation processes in the whole reactions were significantly reduced. Thus, the Fe sites were also the active centers for ETP reaction in OCT-Fe_3_S_4_/PDS system.

**Figure S23.** High-resolution XPS spectra of Fe 2p for fresh and used Fe_3_S_4_ catalysts: (a) FLO-Fe_3_S_4_ and (b) OCT-Fe_3_S_4_.

**Figure S24.** FTIR spectra of fresh and used catalysts: (a) FLO-Fe_3_S_4_ and (b) OCT-Fe_3_S_4_.

**Note:** The stretching vibration peaks at 3436 cm^-1^, 1623 cm^-1^, 1059 cm^-1^ and 570 cm^-1^ were observed in the FTIR spectra of all Fe_3_S_4_ samples, which corresponded to O-H bond, C=O bond, C-S bond and Fe-S bond, respectively.

**Figure S25.** Effect of S^2-^ ion on PDS activation for CLO degradation. Reaction conditions: [PDS] = 1.0 mM, [CLO] = 5 mg/L, and T = 25 ^◦^C.

**Figure S26.** (a-b) Experiments of unsaturated S removed.

**Note:** Solid lines in (a) mean fresh Fe_3_S_4_ catalysts, while dotted lines mean Fe_3_S_4_ catalysts that unsaturated S were removed by AgNO_3_. Since unsaturated S atoms on catalyst can be trapped to form H_2_S, which can react with Ag^+^ to form Ag_2_S. Thus, AgNO3 was used to further confirm the existence of S defect. In detail, 50 mg of Fe_3_S_4_ catalysts were immersed in 1 mM AgNO_3_ solution for full stirring. Then, Fe_3_S_4_ catalysts without the unsaturated S atoms were sucked out using magnet to separate Ag_2_S, and then used to activate PDS for degrading CLO.

**Figure S27.** EPR spectra of fresh and used catalysts: (a) FLO-Fe_3_S_4_ and (b) OCT-Fe_3_S_4_.

**Figure S28.** The Fe(Ⅱ) leaching amount of different catalyst during PDS activation. Reaction conditions: [Catalysts] = 0.1 g/L, [PDS] = 1.0 mM, initial pH = 3.40, [CLO] = 5 mg/L, and T = 25 ^◦^C.

**Figure S29.** Scavenging effects of phenanthroline on CLO degradation in the FLO-Fe_3_S_4_ and OCT-Fe_3_S_4_/PDS systems. Reaction conditions: [Catalysts] = 0.1 g/L, [PDS] = 1.0 mM, initial pH = 3.40, [CLO] = 5 mg/L, [phenanthroline] = 0.5 mM, and T = 25 ^◦^C.

**Figure S30.** (a) CLO degradation efficiencies via PDS activation by filtrates obtained through separating FLO-Fe_3_S_4_ and OCT-Fe_3_S_4_ catalysts. (b) Effect of Fe(Ⅱ) ion on PDS activation. Reaction conditions: [Catalysts] = 0.1 g/L (a), [PDS] = 1.0 mM, initial pH = 3.40, [CLO] = 5 mg/L, and T = 25 ^◦^C.

**Note:** The preparation of filtrates: 4 mg FLO-Fe_3_S_4_ (or OCT-Fe_3_S_4_) was added to the reaction solution ([PDS] = 1 mM, [CLO] = 5 mg/L) for stirring 2 minutes. Afterward, the FLO-Fe_3_S_4_ (or OCT-Fe_3_S_4_) catalyst was separated from solution through 0.22 μm PES membranes. The filtrate contained abundant dissolved Fe ions, which could construct a homogeneous PDS activation system for further CLO degradation.

**Figure S31.** SEM images of fresh and used catalysts: (a-d) FLO-Fe_3_S_4_ and (e-h) OCT-Fe_3_S_4_.

**Figure S32.** Zeta potentials of FLO-Fe_3_S_4_ and OCT-Fe_3_S_4_ at different pH.

**Figure S33.** The total Fe leaching concentrations of (a) FLO-Fe_3_S_4_ and (b) OCT-Fe_3_S_4_ under different pH conditions. Reaction conditions: [Catalysts] = 0.1 g/L, [PDS] = 1.0 mM, initial pH = 3.40, [CLO] = 5 mg/L, and T = 25 ^◦^C.

**Figure S34.** The chemical structure of CLO.

**Figure S35.** Fukui index of CLO.

**Supplementary Tables**

**Table S1.** Comparison of various catalysts in recently reported PDS activation processes.

| Catalyst | Reaction condition | Contaminant  (mg/L) | Removal (%) | k_obs_ (min^-1^) | TOF (min^-1^. M^-1^) | Reference |
| --- | --- | --- | --- | --- | --- | --- |
| FLO-Fe_3_S_4_ | [Catalyst] = 0.1 g/L, [PDS] = 1 mM | Clothianidin  (5 mg/L) | 100.0  (20 min) | 0.4270 | 21.35 | This work |
| OCT-Fe_3_S_4_ | [Catalyst] = 0.1 g/L, [PDS] = 1 mM | Clothianidin  (5 mg/L) | 100.0  (20 min) | 0.0968 | 4.84 | This work |
| 6PA-Ni_4_ | [Catalyst] = 0.15 g/L, [PDS] = 1 mM | Tetracycline  (20 mg/L) | 94.5  (180 min) | 0.0317 | 4.23 | [15] |
| ZnCl_2_-SPI | [Catalyst] = 0.15 g/L, [PDS] = 1 mM | Acetaminophen  (40 mg/L) | 96.0  (5 min) | 0.0180 | 4.80 | [16] |
| S, N-Fe@BC | [Catalyst] = 0.75 g/L, [PDS] = 5 mM | Tetracycline  (20 mg/L) | 90.6  (60 min) | 0.0322 | 0.17 | [17] |
| CoFe_2_O_4_/CFB | [Catalyst] = 0.8 g/L, [PDS] = 3.3 mM | Polystyrene nanoplastics  (100 mg/L) | 88.3  (30 min) | 0.0630 | 2.39 | [18] |
| FMS | [Catalyst] = 0.2 g/L, [PDS] = 3 mM | Monochlorobenzene  (45 mg/L) | 97.8  (90 min) | 0.0410 | 3.08 | [19] |
| Fe/Mo-CNs | [Catalyst] = 0.3 g/L, [PDS] = 3 mM | Bisphenol S  (5 mg/L) | 100.0  (70 min) | 0.0800 | 0.44 | [20] |
| Fe_3_O_4_@Fe^0^-β-CD | [Catalyst] = 0.5 g/L, [PDS] = 5 mM | Naphthalene  (10 mg/L) | 100.0  (6 min) | 0.5710 | 2.28 | [21] |
| MgFe_2_O_4_/BC | [Catalyst] = 0.3 g/L, [PDS] = 3.4 mM | Tetracycline  (30 mg/L) | 94.7  (90 min) | 0.0288 | 0.86 | [22] |
| CuFe_2_O_4_/MXene | [Catalyst] = 0.5 g/L, [PDS] = 2 mM | Atrazine  (10 mg/L) | 97.5  (120 min) | 0.0322 | 0.32 | [23] |
| CuO/Cu_2_(V_2_O_7_)/V_2_O_5_ | [Catalyst] = 1 g/L, [PDS] = 1 mM | Ciprofloxacin  (10 mg/L) | 92  (40 min) | 0.0700 | 0.70 | [24] |

**Note:** The turnover frequency (TOF) was calculated by dividing the observed rate constant of organic contaminants by the catalyst dosage and cocatalyst concentration followed by multiplying the organic contaminant concentration.

$$\text{TOF = }\frac{\text{k}\text{obs}\text{ (min}\text{-1}\text{)}}{\text{Catalyst }\left( \text{mg/L} \right)\text{×PDS (M)}}\text{ × Contaminants (mg/L)}$$

**Table S2.** The identified intermediates of CLO by UPLC−QTOF−MS in the FLO-Fe_3_S_4_/PDS and OCT-Fe_3_S_4_/PDS systems.

(a) Positive ion mode

| Code | Chemical structure | RT (min) | MFG formula | m/z [M+H]^+^ | △ (ppm) |
| --- | --- | --- | --- | --- | --- |
| CLO |  | 7.057 | C_6_H_8_ClN_5_O_2_S | 250.1778 | -0.36 |
| TP-102 |  | 0.935 | C_3_H_3_NOS | 102.1278 | -0.27 |
| TP-119 |  | 1.297 | C_2_H_6_N_4_O_2_ | 119.0563 | -0.07 |
| TP-130 |  | 3.922 | C_4_H_7_N_3_O_2_ | 130.1591 | -0.24 |
| TP-131 |  | 4.876 | C_4_H_9_N_3_O_2_ | 131.9668 | 1.01 |
| TP-134 |  | 0.905 | C_4_H_4_ClNS | 134.1175 | -0.24 |
| TP-149 |  | 7.674 | C_4_H_5_ClN_2_S | 149.0233 | 0.58 |
| TP-205 |  | 7.68 | C_6_H_9_ClN_4_S | 205.0859 | -0.41 |
| TP-206 |  | 4.874 | C_6_H_8_ClN_3_OS | 206.0161 | 0.1 |
| TP-221 |  | 5.412 | C_6_H_9_ClN_4_OS | 221.0072 | 0.03 |

(b) Negative ion mode

| Code | Chemical structure | RT (min) | MFG formula | m/z [M-H]^-^ | △ (ppm) |
| --- | --- | --- | --- | --- | --- |
| CLO |  | 4.872 | C_6_H_8_ClN_5_O_2_S | 248.0012 | 0.44 |
| TP-117 |  | 1.286 | C_2_H_6_N_4_O_2_ | 117.0416 | 0.09 |
| TP-233 |  | 4.429 | C_6_H_6_ClN_3_O_3_S | 233.9854 | -0.35 |

**Table S3.** The HPLC analysis detection parameters for different pollutants.

| Contaminants | Mobile phase | Flow rate  (mL/min) | Column temperature  (℃) | Detection wavelength  (nm) |
| --- | --- | --- | --- | --- |
| CLO | Methanol: Ultrapure water  (50%:50%, v/v) | 0.500 | 25 | 254 |
| THM | Methanol: Ultrapure water  (50%:50%, v/v) | 0.500 | 25 | 254 |
| IMI | Methanol: Ultrapure water  (50%:50%, v/v) | 0.500 | 25 | 270 |
| BPA | Methanol: Ultrapure water  (70%:30%, v/v) | 0.500 | 25 | 280 |
| BPS | Methanol: Ultrapure water  (70%:30%, v/v) | 0.500 | 25 | 260 |
| BPAF | Methanol: Ultrapure water  (70%:30%, v/v) | 0.500 | 25 | 248 |

**Supplementary References**

[1] G. Kresse, J. Furthmüller, Efficient Iterative Schemes for Ab Initio Total-Energy Calculations Using a Plane-Wave Basis Set, Phys. Rev. B 54 (1996) 11169–11186.

[2] P. E. Blöchl, Projector augmented-wave method, Phys. Rev. B 50 (1994) 17953–17979.

[3] J. P. Perdew, K. Burke, M. Ernzerhof, Generalized Gradient Approximation Made Simple, Phys. Rev. Lett. 77 (1996) 3865–3868.

[4] M. Methfessel, A. T. Paxton, High-precision sampling for Brillouin-zone integration in metals, Phys. Rev. B (1989) 3616–3621.

[5] S. Grimme, J. Antony, S. Ehrlich, H. Krieg, A consistent and accurate ab initio parametrization of density functional dispersion correction (DFT-D) for the 94 elements H-Pu, J. Chem. Phys. (132) 2010 154104.

[6] Gaussian 16, Revision A.01, M. J. Frisch, G. W. Trucks, et al, Gaussian, Inc., Wallingford CT, (2016). <https://gaussian.com>

[7] P.J. Stephens, F.J. Devlin, C. F Chabalowski, M.J. Frisch, Ab Initio Calculation of Vibrational Absorption and Circular-Dichroism Spectra Using Density-Functional Force-Fields, J. Phys. Chem. 98 (1994) 11623-11627.

[8] S. Grimme, J. Antony, S. Ehrlich, H. Krieg, A consistent and accurate ab initio parametrization of density functional dispersion correction (DFT-D) for the 94 elements H-Pu, J. Chem. Phys. 132 (2010) 154104.

[9] P. C. Hariharan, J. A. Pople, The influence of polarization functions on molecular orbital hydrogenation energies, Theor. Chim. Acta. 28 (1973) 213-222.

[10] W. J. Hehre, R. Ditchfield, J. A. Pople, Self—consistent molecular orbital methods. XII. Further extensions of Gaussian—type basis sets for use in molecular orbital studies of organic molecules, Chem. Phys. 56(5) (1972) 2257-2261.

[11] R. B. J. S. Krishnan, J. S. Binkley, R. Seeger, J. A. Pople, Self‐consistent molecular orbital methods: A basis set for correlated wave functions, J. Phys. Chem. 72(1) (1980) 650-654.

[12] R. G. Parr, W. Yang, Density functional approach to the frontier-electron theory of chemical reactivity, J. Am. Chem. Soc. 106(14) (1984) 4049-4050.

[13] T. Lu, F. Chen, Multiwfn: a multifunctional wavefunction analyzer, J. Comput. Chem. 33 (2012) 580-592.

[14] J. Hu, X. Xu, Y. Ji, H. Zhu, Y. Zhou, M. Zhu, Y. Huang, Role of Mn(III) intermediates in the degradation of carbamazepine via peroxymonosulfate activation by manganese single-atom catalysts: Radical and non-radical synergistic effects, Appl. Catal. B Environ., 373 (2025) 125337.

[15] X. Wang, W. Li, J. Zhang, S. Jiang, G. Zhang, C. Bai, S. Wang, Q. Zhao, L. Lv, The critical roles of surface-bound radicals in the nickel phosphide/biochar-persulfate catalytic oxidation system for tetracycline removal: The synergistic catalysis between nickel phosphides and biochar, Chem. Eng. J. 491 (2024) 151915.

[16] H. Yang, G. Choi, Y. Jeong, I. Cho, S. Park, C. Lee, Enhancing acetaminophen removal through persulfate activation with ZnCl_2_-SPI biochar: A study on reactive oxygen species contribution according to acetaminophen concentration, Chem. Eng. J. 496 (2024) 154065.

[17] J. Liu, J. Zhu, Q. Gao, X. Zeng, Q. Zeng, J. Xiong, G. Zhang, Y. Niu, H. Xie, Accelerate degradation of tetracycline via persulfate activation over S, N-Fe co-doped banana peel biochar: The role of pyrolysis temperature and ^1^O_2_ evolution, Chem. Eng. J. 509 (2025) 161425.

[18] M. Qaretapeh, S. Kouchakipour, M. Hosseinzadeh, K. Dashtian, Cuttlefish Bone-Supported CoFe_2_O_4_ nanoparticles enhance persulfate Fenton-like process for the degradation of polystyrene nanoplastics, Chem. Eng. J. 490 (2024) 151833.

[19] Z. Feng, J. Yu, Z. Yang, D. Liu, J. Xu, Y. Ning, F. Jiang, S. Yang, Y. Li, Fe-Mn bimetallic sulfide enhanced persulfate activation for monochlorobenzene degradation in groundwater: Performance, mechanism and applicability, Chem. Eng. J. 513 (2025) 162790.

[20] Z. Liu, S. Pan, F. Xu, Z. Wang, C. Zhao, X. Xu, B. Gao, Q. Li, Revealing the fundamental role of MoO_2_ in promoting efficient and stable activation of persulfate by iron carbon based catalysts: Efficient Fe^2+^/Fe^3+^ cycling to generate reactive species,

Water Res. 225 (2022) 119142.

[21] Y. Wang, M. Wang, Q. Xie, X. Cai, Developments of cyclodextrin-coated Fe_3_O_4_@Fe^0^ nanoparticles to both efficiently activate persulfates and rapidly access hydrophobic PAHs in water, Chem. Eng. J. 468 (2023) 143510.

[22] L. Wang, X. Lu, G. Chen, Y. Zhao, S. Wang, Synergy between MgFe_2_O_4_ and biochar derived from banana pseudo-stem promotes persulfate activation for efficient tetracycline degradation, Chem. Eng. J. 468 (2023) 143773.

[23] Z. Chen, J. Guo, Z. Kuang, W. Chen, Y. Yang, Q. Zhou, Accordion-like MXene-supported CuFe_2_O_4_ actuates persulfate activation for enhanced atrazine degradation: Radical mechanism, DFT calculations and potential toxicity evaluation, Chem. Eng. J. 500 (2024) 157179.

[24] Y. Xue, M. Kamali, X. Yu, L. Appels, R. Dewil, Novel CuO/Cu_2_(V_2_O_7_)/V_2_O_5_ composite membrane as an efficient catalyst for the activation of persulfate toward ciprofloxacin degradation, Chem. Eng. J. 455 (2023) 140201.
